# Sugarcane cultivation practices modulate rhizosphere microbial community composition and structure

**DOI:** 10.1038/s41598-022-23562-6

**Published:** 2022-11-10

**Authors:** Ana Paula Corrêa Moneda, Lucas Amoroso Lopes de Carvalho, Luis Guillermo Teheran-Sierra, Michelli Inácio Gonçalves Funnicelli, Daniel Guariz Pinheiro

**Affiliations:** 1 Laboratory of Bioinformatics, Department of Agricultural, Livestock and Environmental Biotechnology, School of Agricultural and Veterinary Sciences, São Paulo State University (UNESP), Jaboticabal, SP 14884-900 Brazil; 2 Graduate Program in Agricultural and Livestock Microbiology, School of Agricultural and Veterinary Sciences, São Paulo State University (UNESP), Jaboticabal, SP Brazil

**Keywords:** Computational biology and bioinformatics, Ecology, Microbiology

## Abstract

Sugarcane (*Saccharum* spp.) represents a crop of great economic importance, remarkably relevant in the food industry and energy supply chains from renewable sources. However, its conventional cultivation involves the intensive use of fertilizers, pesticides, and other agrochemical agents whose detrimental effects on the environment are notorious. Alternative systems, such as organic farming, have been presented as an environmentally friendly way of production. Still, the outcomes of different cropping systems on the microbiota associated with sugarcane—whose role in its health and growth is crucial—remain underexplored. Thus, we studied the rhizospheric microbiota of two adjacent sugarcane fields, which differ in terms of the type of farming system. For this, we used the sequencing of taxonomic markers of prokaryotes (gene 16S rRNA, subregions V3–V4) and fungi (Internal transcribed spacer 2) and evaluated the changes caused by the systems. Our results show a well-conserved microbiota composition among farming systems in the highest taxonomic ranks, such as phylum, class, and order. Also, both systems showed very similar alpha diversity indices and shared core taxa with growth-promoting capacities, such as bacteria from the *Bacillus* and *Bradyrhizobium* genera and the fungal genus *Trichoderma*. However, the composition at more specific levels denotes differences, such as the separation of the samples concerning beta diversity and the identification of 74 differentially abundant taxa between the systems. Of these, 60 were fungal taxa, indicating that this microbiota quota is more susceptible to changes caused by farming systems. The analysis of co-occurrence networks also showed the formation of peripheral sub-networks associated with the treatments—especially in fungi—and the presence of keystone taxa in terms of their ability to mediate relationships between other members of microbial communities. Considering that both crop fields used the same cultivar and had almost identical soil properties, we conclude that the observed findings are effects of the activities intrinsic to each system and can contribute to a better understanding of the effects of farming practices on the plant microbiome.

## Introduction

Sugarcane (*Saccharum* spp.) stands out in Brazil as one of the most important socioeconomic crops. The agro-industrial system related to sugarcane processing in biorefineries is responsible for supplying the market with ethanol biofuels and sugar. Furthermore, sugarcane biorefineries can also perform the co-production of large amounts of useful products from wastes, such as bagasse, molasses, cane trash, filter mud, and vinasse. All of them have significant value-added based on the concept of circular bioeconomy and sustainability production^1^. These concepts are congruent with the consumer’s concerns for healthier lifestyles and environmental care^2^.

In order to meet rising consumer demands for higher-quality products from a more sustainable production, there is a growing interest in the development and adoption of agricultural models that aims to conserve and enhance the quality of the soil leading to higher yields but taking into account the protection of the local environment and ecosystem services^3^. These agroecological practices are the basis of organic farming systems, which prohibit the use of synthetic inputs, such as pesticides or fertilizers^4^. Organic farming highlights the essential role of humus and organic matter for soil fertility and plant nutrition^5,6^. The great challenge for organic production is to achieve higher crop yield stability^7^. To overcome that, it is necessary a deep and integrated understanding of climate and biogeochemical cycles, pollination, soil structure and protection, water absorption, and biological interactions, among other processes. In this context, the soil microbiota can also play a fundamental role^8^, especially those that inhabit the rhizosphere^9^ and colonize the plant tissues^10^. Therefore, the soil and plant-associated microbiota has become the target of studies to identify the driving factors that shape microbial assemblage composition and structuration^11^.

In this sense, culture-independent methods for the investigation of plant microbiota, such as the sequencing of amplicons (or metabarcoding) from taxonomic marker regions, such as the 16S rRNA gene^12^ and the Internal Transcribed Spacer (ITS) region^13^, for prokaryotes and fungi, respectively, have enabled to advance the understanding of microbial communities and their relationships^14,15^. The research on sugarcane-associated microbiota aims to investigate the microbial diversity reservoir still unexploited to acquire knowledge about its role in modulation of plant development, pathogen defense, nutrient uptakes, and stress resistance^16^. This is essential to constitute the foundation for the development of solutions to equilibrate higher productivity with sustainability for this crop. Recent studies indicate that different sugarcane genotypes can shape the associated microbiota by changing from keystone species to the richness of bacterial and fungal communities^17,18^.

The sugarcane rhizosphere is a very relevant ecosystem for deepening knowledge about the plant-associated microbiota^4^. It is characterized as an environment of interaction events between the plant and microorganisms, through intensive chemical signaling exchange^19^⁠. The assemblage of microorganisms in there are part of a complex interaction network, allowing the plant to modulate the microbiome for its own benefit, through the selection of microorganisms with suitable feature to meet its needs, favoring its healthy growth and development^9^. Among these, there are the so-called plant growth promoters, which provide an increase in absorption of minerals, mobilization of nutrients, and a decrease in pathogens activity^19,20^.

Few studies have comparatively evaluated the impact of the organic versus conventional farming systems on the structure and composition of the rhizosphere microbiota in agricultural crops, especially on sugarcane^21,22^. There is evidence that organic agriculture has positive effects on the microbial community, with increased richness and diversity^23,24^. On the other hand, recent advances show that the practice of sugarcane monoculture following the precepts of conventional fertilization points to a significant impact on its associated microbiota in the short and long term^25,26^. These impacts include the depletion of beneficial taxa, such as *Rhizobium* and *Sphingomonas*, while potentially phytopathogenic genera are enriched^26^. However, by comparing organic and conventional systems, Orr et al.^27^ did not obtain any results that suggest significant effects on microbial communities, concluding that the environmental and chemical variables of the soil are the ones that really govern the present biodiversity.

To evaluate and compare the microbial community of the sugarcane rhizosphere under two contrasting cropping systems, those being organic and conventional, we adopted the metabarcoding approach, which allowed us to find shifts in its composition, diversity, and structuring.

## Materials and methods

### Experimental design and samples collection

**Figure 1:**
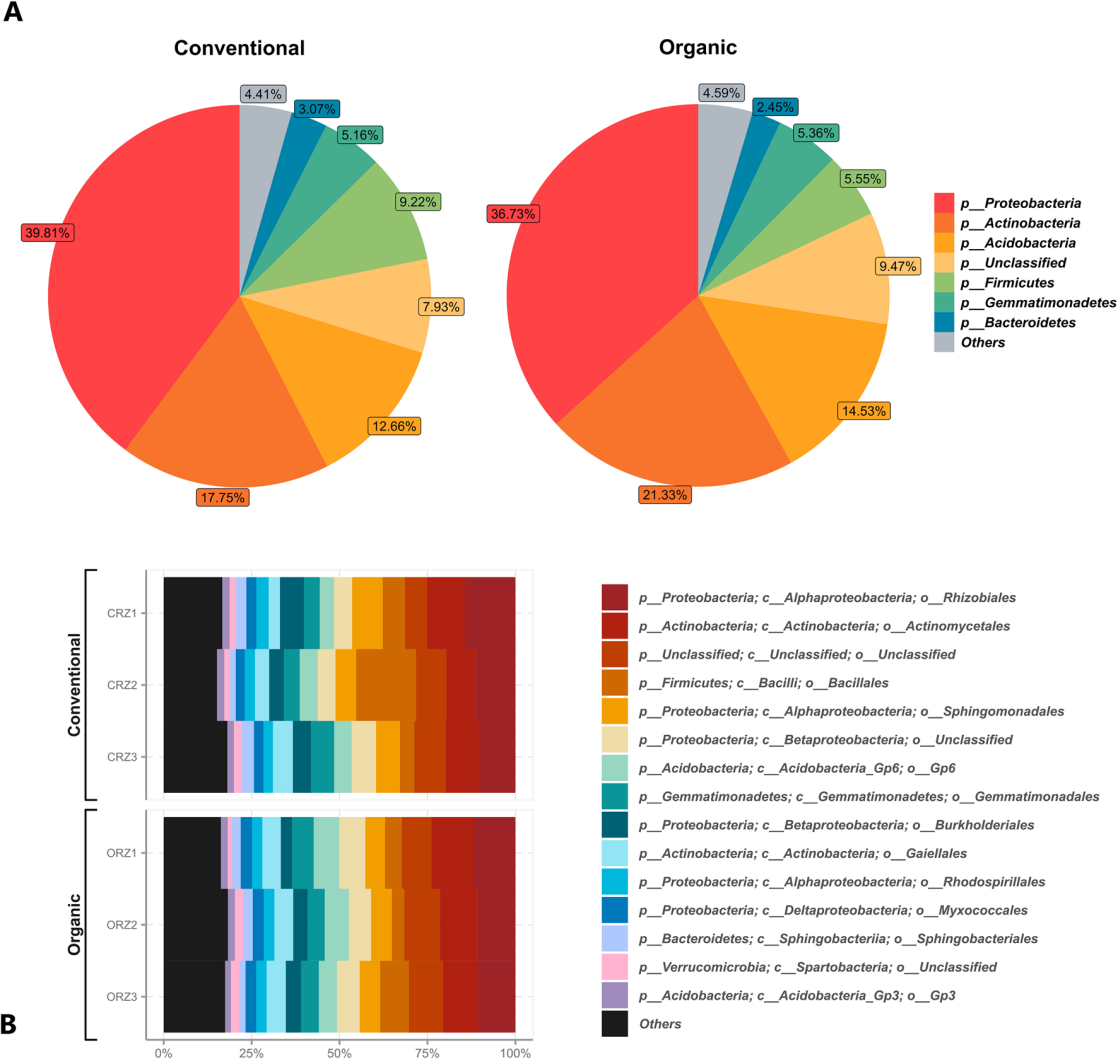
Distribution of the 8 most abundant prokaryotic phyla (**A**) and distribution of the 15 most abundant bacterial orders in the sugarcane rhizosphere of the organic (ORZ) and conventional (CRZ) farming systems (**B**).

The sugarcane rhizosphere samples for metagenomic DNA extraction were collected in 2018 between February and March at the São José Farm (organic farming system) and at the São Sebastião Farm (conventional farming system), both in Jaboticabal, São Paulo State, Brazil. The selected sugarcane field stands for sampling were very close (≈100 m; Supplementary Fig. 1), they comprised plants of the same cultivar (CTC9001) in the plant cane cycle and planted at a similar season/date (March 2017). We made the three samplings of rhizosphere material approximately 11 months after planting, near the end of vegetative growth and the beginning of the sugarcane maturation process. We selected the sampled points from a representative portion of the sugarcane fields from equidistant sampling points (≈50 m) (Fig. 1). The samples from the conventional cultivation field were named CRZ, and the samples from the organic cultivation field were named ORZ. The geolocation, environmental conditions, and sample collection dates (Supplementary Table 1).

In the São José Farm, the sugarcane had been cultivated in a conventional system until the year 2000, when the conversion process started. During the samplings, the Farm had the Brazilian national certificate of organic production^28^ and the American certificate from the United States Department of Agriculture (USDA), besides complying with the regulations defined by the European Union, (EC) n. 834/2007^29,30^.

### Field management in the organic or conventional farming systems

The procedures for preparing the soil for planting are similar in the sugarcane field stands under organic or conventional farming (*i.e.*, plowing, harrowing, and subsoiling). In the renovation of the fields, leguminous (peanuts, soybeans, or crotalaria) were planted. After harvesting these crops, the remaining plant material is turned along with the soil in preparation.

In the area of organic cultivation, for the sugarcane field renovation, there was an application of vinasse in the soil, and specifically in the planting furrow, there was supplementation with organic matter under decomposition process, which were obtained from filter pie, confined cattle manure, and also from the native vegetation of the farm. For replanting, fertilization is initiated with liming and phosphating using mineral fertilizers (limestone and phosphate rock). Commercial products for pest biocontrol and bioinoculants for atmospheric nitrogen fixation were used to promote plant healthy growth (Supplementary Table 2).

In the conventional cultivation field, pH soil correction and fertilization were carried out with liming, gypsum, limestone, and NPK (N, P2O5 e K2O) in a proportion of 50-50-25, respectively. In addition, other fertilizers based on organic and inorganic compounds, and insecticides, fungicides, and herbicides were also applied (Supplementary Table 3).

### Sugarcane rhizosphere sampling

The bulk soil around the sugarcane plants was excavated considering a circumference of 0.5 to 0.6 m^2^ in diameter and a depth of 0.5 m., where the roots and soil adhered to the roots were stored in sterile plastic bags with a capacity of 20 L. The samples were stored in a cold box and taken to the laboratory for processing.

The roots were shaken to break the remaining clumps and loosen the excess soil. After this, the soil firmly adhering to the roots was gently brushed out, sieved, fractionated into six (1.5 mL) microtubes, and then frozen in liquid nitrogen and stored at − 80 °C until the DNA extraction.

### Evaluation of physicochemical soil properties

Chemical and physical analysis of bulk soils were performed, considering a pool of 5 soil samples around each sampling point (approximately 2 m radius). For each sampling point, macronutrient analyzes were performed (Potassium [K], Phosphorus [P], Sulfur [S], Magnesium [Mg] and Calcium [Ca]), micronutrients (Boron [B]), Manganese [Mn] , Iron [Fe], Copper [Cu], Zinc [Zn] and Aluminum [Al]), organic matter (OM), as well as analyzes related to soil acidity (pH, potential acidity [H + Al], sum of bases [SB], base saturation [V%], cation exchange capacity [CTC]), Aluminum saturation [m%], according to the methods described by Instituto Agronômico de Campinas – IAC^31^. For granulometric evaluation, the fractions of Clay, Silt and Sand (“Coarse sand” and “Fine sand”) were determined according to the manual of the Brazilian Agricultural Research Corporation—Embrapa^32^.

The mean values for each of the physico-chemical soil properties obtained for sugarcane fields under the two contrasting systems (organic vs. conventional) were statistically compared using the “compare_means” package, the “ggpubr” function (v. 0.4.0)^33^, of the R statistical program (v 4.0.2) ^34^.

### DNA extraction and purification

The extractions of DNA from microbial communities in the rhizosphere soils were performed using the commercial DNeasy PowerSoil Kit (Qiagen®). The manufacturer's standard protocol was used with the modifications made by de Souza et al.^16^, which consisted of heating at 65 °C for 10 min. after addition of reagent C1 and two washing steps with ethanol.

In order to remove contaminants, such as PCR inhibitors, the DNA was submitted to the purification step with Agencourt AMPure XP kit (A63881, Beckman Coulter®), following the manufacturer's protocol with 1.2 × reagent to sample ratio. The DNA samples were quantified by a spectrophotometer (NanoDrop®) and fluorometer (Qubit®) and stored at − 80 °C.

### Amplicon library preparation 16S (Prokaryotes) and ITS (Fungi) and sequencing

To access the rhizosphere prokaryotic communities, PCR amplification products (amplicons) of the 16S rRNA gene were used, targeting the fragment comprising the V3 and V4 hypervariable regions of the gene (primers 319F [5′-ACTCCTACGGGAGGCAGCAG] and 806R [5′-GGACTACHVGGGTWTCTAAT]; 469 bp). To access the rhizosphere fungal communities, the Internal Transcribed Spacers (ITS) were used, and in this case, the target was the ITS2 region (primers ITS9F [5′-GAACGCAGCRAAIIGYGA] and ITS4R [5′-TCCTCCGCTTATTGATATGC]; variable size)^35^.

The amplicon libraries for Next Generation Sequencing (NGS) were constructed in two PCR steps. In the first PCR step, target-specific amplification was performed, which adds the Illumina adapter and 4 to 6 random bases adjacent to the forward and reverse primers to incorporate heterogeneity into the read sequences^16,36^. In the second step, Illumina's N7 and S5 barcodes were incorporated for indexing the reads in the subsequent multiplex sequencing. The PCR steps followed the same protocol described in the Illumina library preparation technical manual for MiSeq, using PCRBIO ULTRA MIX kit for PCRs. The samples were purified with the Agencourt AMPure XP kit (A63881, Beckman Coulter®) and also with the DNA Clean & Concentrator kit—Zymo Research®.

In total, 12 libraries were built, 6 for each of the farming systems (*i.e.* three sample units (replicates) for prokaryotic communities [16S] and three for fungal communities [ITS]). The three sample units correspond to each one of the sampling points (Supplementary Fig. 1).

The sequencing of the 16S and ITS amplicon libraries was performed on the Illumina® MiSeq platform. The amplicon fragments were sequenced using the MiSeq Reagent Kit v.2 (600 cycles) producing 300 bp reads in paired-end mode (2 × 300 bp).

### Data processing of 16S and ITS amplicon libraries

The demultiplexing of the sequence reads was performed using “bcl2fastq” software (v2.20.0.422) (Illumina®) with default settings. The remaining reads, whose barcodes were not identified, were processed with “deML” program (v.1.1.3)^37^ using the *-rgqual 90* and *-wrongness 80* as parameter settings.

The paired-end reads corresponding to the amplicons were merged using the “PEAR” tool (v.0.9.11)^38^, with a minimum overlap of 15 bp. The amplicon sequences corresponding exactly to the V3–V4 regions of the 16S rRNA and ITS2 gene were extracted using the “search_pcr2” command of the “USERCH” toolkit (v.11.0.667)^39^.

For the microbiome analysis, we used the Divisive Amplicon Denoising Algorithm – “DADA2” pipeline (v1.14.1)^40^ to infer and quantify Amplicon Sequence Variants (ASVs). The pipeline was implemented in R (v 4.0.2)^34^.

Both sets of merged read sequences, *i.e.* from the region V3–V4 of the 16S rRNA gene and from the ITS2 region, were filtered using the “filterAndTrim” function with the following parameter settings: *maxN* = *0*, *truncQ* = *2* and *maxEE* = *2*. The error rate per sample was estimated based on the error model using the “learnErrors” function and sequence redundancy was removed using the “derepFastq” function. Finally, the sequences were corrected based on the error models obtained previously with the “dada” function and chimeric sequences were removed using a “removeBimeraDenovo” function.

The taxonomic assignment of ASVs was performed by the RDP Naive Bayesian classifier (Wang et al. 2007) through DADA2 function “assignTaxonomy” with the following parameter settings: *minboot* = *80*, and *refFasta* with the file path corresponding to the suitable reference database, *i.e.* the RDP database training set (v.16)^41^ for the 16S dataset and the UNITE (v.8.2)^13^ database for the ITS2 dataset.

### Microbial communities' diversity

For the alpha-diversity indices were estimated from the absolute counts obtained for ASVs of prokaryotes (mainly bacteria) and fungi of both farming systems were considered. From these data, the parameters of richness (Chao1) and diversity (Shannon and Gini-Simpson indices) were estimated for both datasets. For this, the ASV counts were transformed into a “phyloseq” object (package “phyloseq”)^42^ and subsequently submitted to the “alpha” function of the R package “microbiome”^43^. The diversity measures were statistically compared using the “compare_means” function of the R package “ggpubr” (v. 0.4.0)^33^, using the Wilcoxon non-parametric test for comparing means, and considering a *p*-value ≤ 0.1 as statistically significant.

For the beta-diversity analysis, the absolute counts for ASVs in both datasets were transformed to compositional matrices (*i.e.* normalization by TSS – Total Sum Scaling, or relative abundances), through the “transform” function of the “microbiome” package. From the transformed values, we calculated the distances of the Bray–Curtis dissimilarities (“distance” function of the “phyloseq” package). The distance matrices were used in a statistical comparison between the farming systems by a PERMANOVA (Permutational Multivariate Analysis of Variance) analysis, using the “adonis” function of the “vegan” package^44^, considering a *p*-value ≤ 0.1 as statistically significant. Then, the matrices were used to obtain a dendrogram, resulting from the hierarchical grouping of the samples, and provided as input for a Principal Coordinate Analysis (PCoA).

### Core microbiome

The recognition of the core microbiome of sugarcane rhizosphere (*i.e.* the one common for the farming systems) was done through the identification of microorganisms (prokaryotes or fungi) with the high prevalence and abundance in all the samples, independently of the label corresponding to the farming system. For this, we considered those microorganisms present in at least 90% of the samples, with a minimum relative abundance of 1%. The calculations and visualizations of the core microbiome were obtained through the “plot_core” function of the R package “microbiome”.

#### Differential abundance analysis

The identification of taxa, from each taxonomic level, which are present in significantly different abundances between farming systems, was done using the “DESeq2” approach^45^. For this, we submit the prokaryotes and fungi datasets to the “MicrobiomeAnalyst” platform^46^. In the web platform, the datasets with ASV counts were normalized using Total Sum Scaling (TSS), and also provided as input for DESeq2 analysis, in which the Wald test was performed to evaluate statistical significance, considering a *p*-value ≤ 0.01 as statistically significant.

#### Predictive functional profiling of microbial communities

The functional capabilities of the microbiomes from the sugarcane rhizosphere under the considered farming systems were predicted using the “PICRUSt2” program (v.2.3.0-b)^47^. For this, we used the metabolic pathway database “MetaCyc” (Caspi 2006) as a reference for the functional annotations. A comparison of the annotated pathways between the datasets of corresponding farming systems was performed to identify enriched metabolic pathways associated with one of them. For this purpose, the “STAMP” program (v.2.1.3)^48^ was used for the application of White’s non-parametric t-test to compare the means, considering a *p*-value ≤ 0.05 as statistically significant.

#### Co-occurrence networks

The co-occurrence networks of the identified genera of fungi and prokaryotes were elaborated based on Pearson's correlation coefficients (r). The coefficients were obtained from the normalized ASVs and were forwarded to the “Correlation Analysis”. The results were filtered using the following criteria: absolute correlation threshold of 0.5 (r ≥ 0.5 or r ≤ − 0.5) and *p*-value ≤ 0.05. Subsequently, based on the filtered results, the relationships between each genus in addition to their respective correlation coefficients were suitably formatted and used as input for the “Cytoscape” program (v.3.8.0)^49^. In this program, in addition to visual representations, the topological network parameters, such as the measures of centrality, were obtained by using the Cytoscape plugin “NetworkAnalyzer”^50^.

## Results

### Physicochemical soil properties

The physicochemical analysis of the soils showed few significant differences between the crops. Conventional cultivation was slightly more acidic, with lower pH, sum and base saturation values, in contrast to the higher percentage of aluminum saturation when compared to the organic cultivation. All values obtained in soil analysis, as well as statistical comparisons, can be found in Supplementary Table 4.

### Amplicon Sequence Variants of 16S and ITS datasets

The high-throughput sequencing of sugarcane rhizosphere soil using the Illumina MiSeq instrument resulted in a total of 329,685 paired-end reads from the 16S rRNA gene (V3–V4 region), with an approximate average of 55,000 pairs per library. Of these, about 227,607 were successfully assigned as ASVs. For the ITS2 amplicons, the sequencing resulted in a total of 311,269 paired-end reads, an average of 51,000 pairs per library, of which 276,069 were successfully assigned as ASVs. Among ASVs, 80,465 and 183,705 received a taxonomic assignment at genus level, respectively, for 16S and ITS datasets. (Supplementary Table 5).

### Taxonomic composition and ecological measures

In the prokaryotic taxonomic composition, most of the most abundant taxa belongs to the phylum Proteobacteria in both management systems, followed by the phyla Actinobacteria, Acidobacteria, Firmicutes, Gemmatimonadetes and Bacteroidetes (Fig. 1A). It is notable the presence of groups categorized as unclassified, grouping taxa in lower abundances, which probably are unknown in the reference database or have sequences with insufficient evidence for the taxonomic definition. Considering the taxonomic level of order, among the most abundant, Rhizobiales, Actinomycetales, Bacillales, and Sphingomonadales stand out (Fig. 1B).

**Figure 2:**
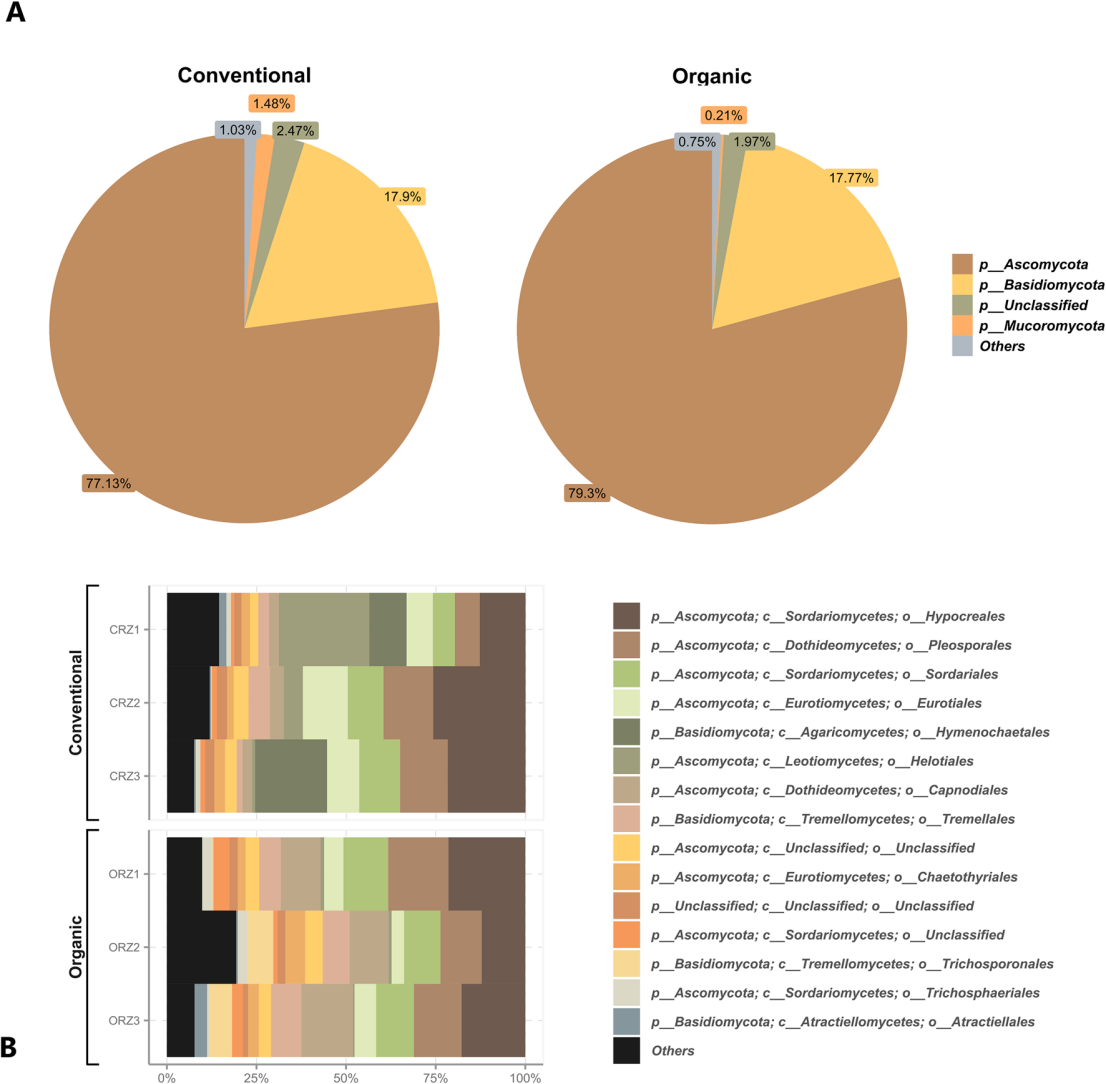
Distribution of the 5 most abundant fungal phyla (**A**) and distribution of the 15 most abundant fungal orders in the sugarcane rhizosphere of the organic (ORZ) and conventional (CRZ) farming systems (**B**).

In the fungal taxonomic composition, the most abundant phyla are Ascomycota and Basidiomycota, followed by Mucoromycota, with relatively low abundance (Fig. 2A). Among the most abundant orders, Hypocreales, Perosporales, and Sordariales stand out (Fig. 2B).

### Richness and diversity

**Figure 3:**
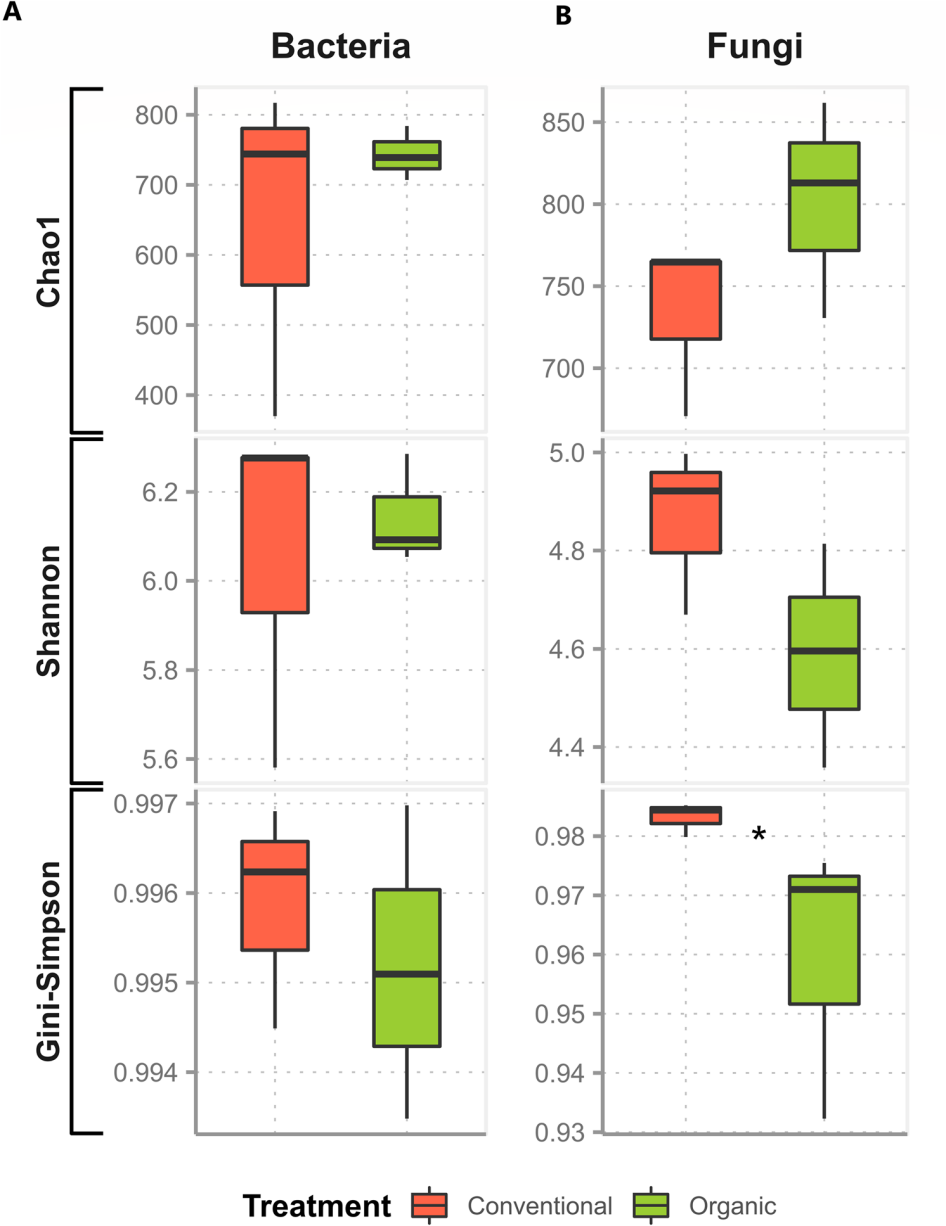
Alpha diversity indices for the 16S (Bacteria) and ITS (Fungi) datasets of the organic and conventional sugarcane rhizosphere soil. The measures were statistically compared using the Wilcoxon nonparametric test of means, considering a *p*-value ≤ 0.1. as statistically significant (*).

The richness and diversity indices of taxa in the prokaryotes dataset in both farming systems did not show significant differences, through the comparison of means (*p*-value > 0.1) (Fig. 3; Supplementary Table 6). Regarding the fungal dataset, there was only one difference in diversity, considering the Gini-Simpson index (*p*-value ≤ 0.1), pointing to a higher diversity in the conventional system. The other indices showed no significant differences between the farming systems, considering the same descriptive level of statistical significance (Fig. 3; Supplementary Table 6).

### Beta diversity

**Figure 4:**
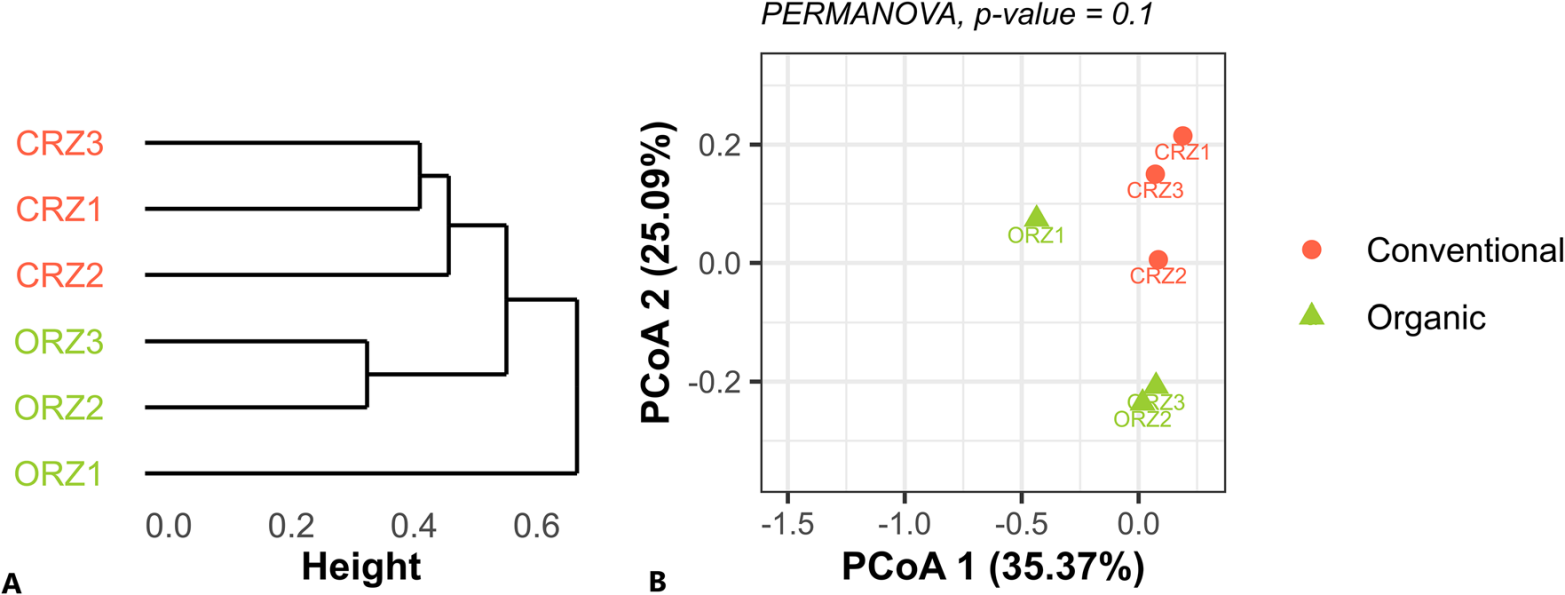
Dendrogram resulting from the hierarchical grouping of 16S rRNA samples from rhizospheric soil (**A**). Principal Coordinate Analysis (PCoA), based on the Bray–Curtis index of 16S rRNA samples from rhizospheric soil (**B**). Distance matrices were used for statistical comparison between the systems by a PERMANOVA analysis, considering a *p*-value ≤ 0.1.

In the two-dimensional Principal Coordinate Analysis (PCoA) plot, based on the Bray–Curtis index (Fig. 4), there was a significant description of the system through a projection of prokaryotic taxa (16S), without major loss of information, preserving 60.46% of the variance in the data. The PcoA 1 index describes most of the variation (accounting for 35.37%). One of the samples from the organic system appears far from the others of the same group, resembling samples from the conventional system, suggesting that it is an outlier. However, even considering this discrepant sample, it is possible to notice the significant separation of the bacterial compositions concerning the farming system factor (PERMANOVA; *p*-value ≤ 0.1).

**Figure 5:**
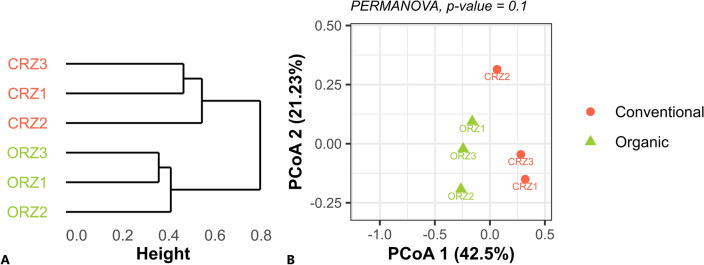
Dendrogram resulting from the hierarchical grouping of ITS samples of rhizospheric soil (**A**). Principal Coordinate Analysis (PCoA), based on the Bray–Curtis index of ITS samples of rhizospheric soil (**B**). Distance matrices were used for statistical comparison between the systems by a PERMANOVA analysis, considering a *p*-value ≤ 0.1.

The PCoA for the fungal dataset (ITS) reveals that the samples present a clustering profile similar to that observed for the prokaryotic dataset, i.e., the samples from the organic and conventional systems are well defined and showed differences between the managements (Fig. 5). The data projection for the fungi dataset preserves 60% of variance in the data, thus enabling a significant description of the system, without major loss of information. The descriptive level of significance by the PERMANOVA analysis was 0.1. The case is similar to the evaluation made for the prokaryotic dataset, thus also representing a significant difference between the systems.

### The core microbiome of sugarcane rhizosphere

**Figure 6:**
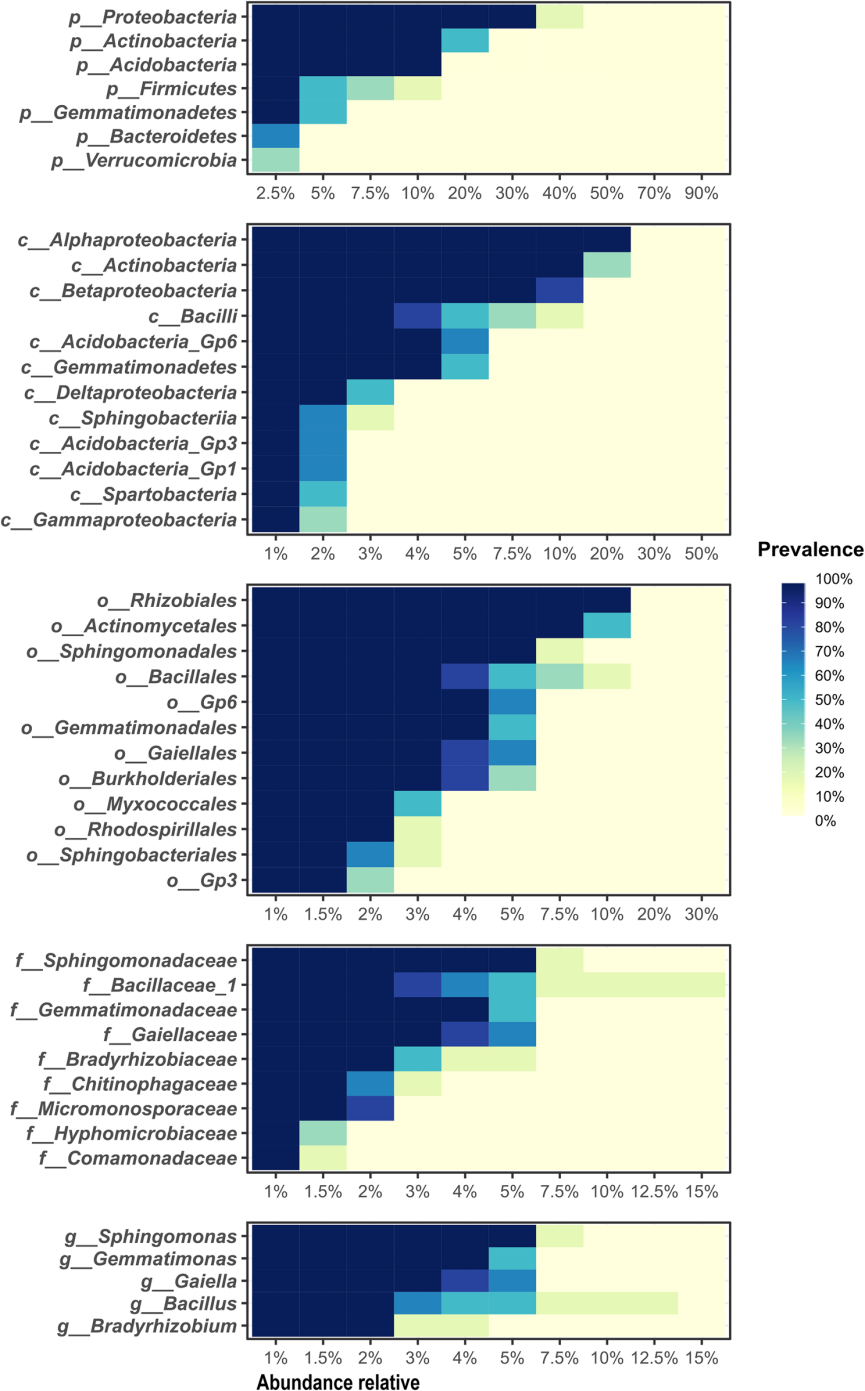
Core microbiome analysis of sugarcane rhizosphere. High prevalence prokaryotic taxa (> 90%) in the rhizosphere samples, regardless of the farming system, where the blue color indicates the prevalence of taxa in the samples and the horizontal percentage represents the relative abundance of each taxon in the respective sample. The taxonomic level can be identified by the prefixes: “p” (phylum), “c” (class), “o” (order), “f” (family), and “g” (genus).

For the 16S rRNA data, 7 phyla, 12 classes, 12 orders, 9 families and 5 genera were considered to belong to the essential microbiome (Fig. 6). Among these, the phylum Proteobacteria stands out, with a relative abundance of 35% in all samples, followed by Actinobacteria, with a relative abundance of 15% in the samples.

**Figure 7:**
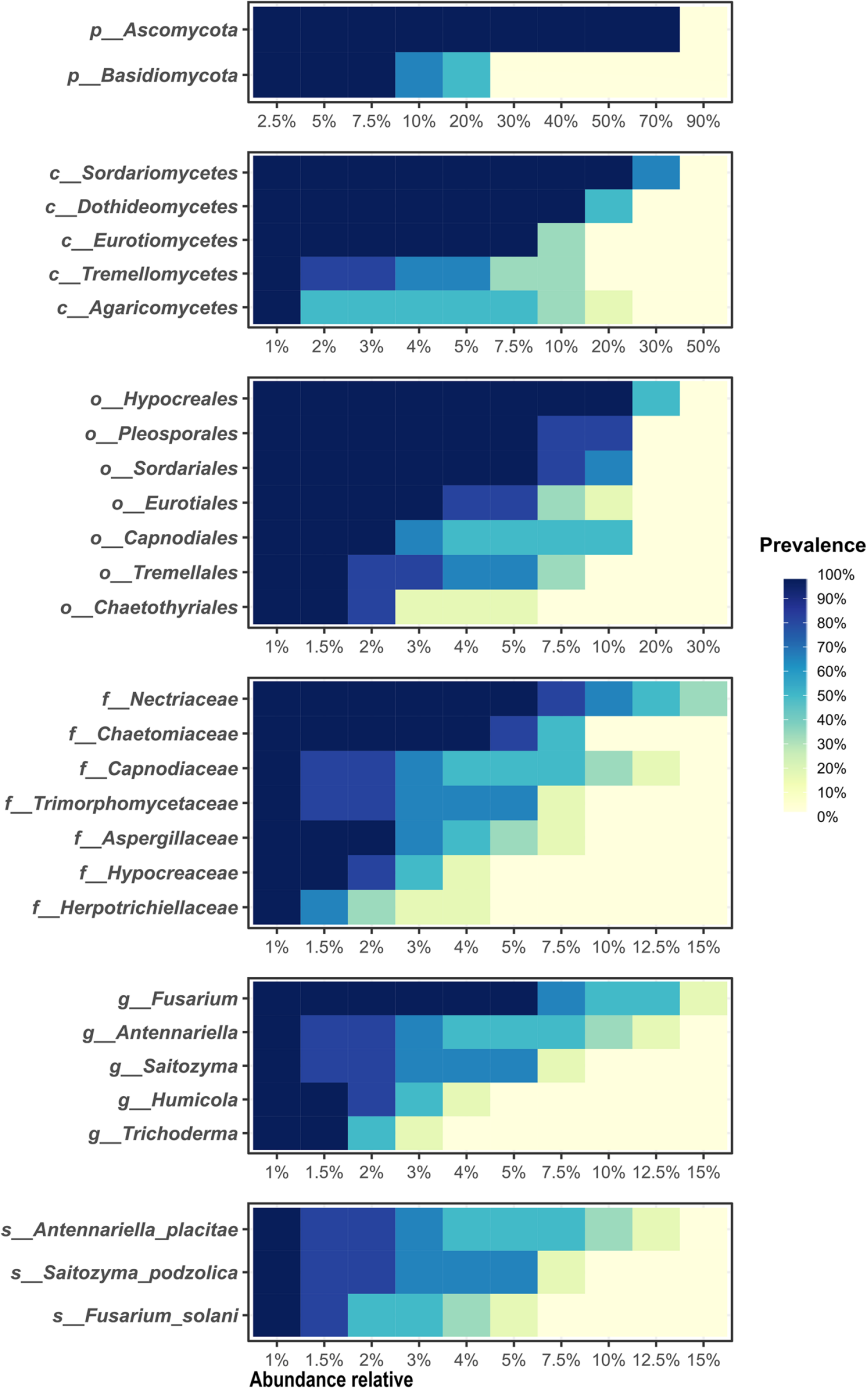
Core rhizosphere sugarcane microbiome analysis. High prevalence fungal taxa (> 90%) in the rhizosphere samples, regardless of the farming system, where the blue color indicates the prevalence of taxa in the samples and the horizontal percentage represents the relative abundance of each taxon in the respective sample. The taxonomic level can be identified by the prefixes: “p” (phylum), “c” (class), “o” (order), “f” (family), and “g” (genus).

The core fungal microbiome comprised 2 phyla, 5 classes, 7 orders, 7 families, 5 genera and 3 species (Fig. 7). The most prevalent essential phylum was Ascomycota, with a relative abundance of 80% in all samples. The phylum with the lowest prevalence was Basidiomycota, with a relative abundance of 8% in the samples. Its only representative was the species *Saitozyma podzolica*.

### Differential abundance of taxa considering the contrasting farming systems

**Figure 8:**
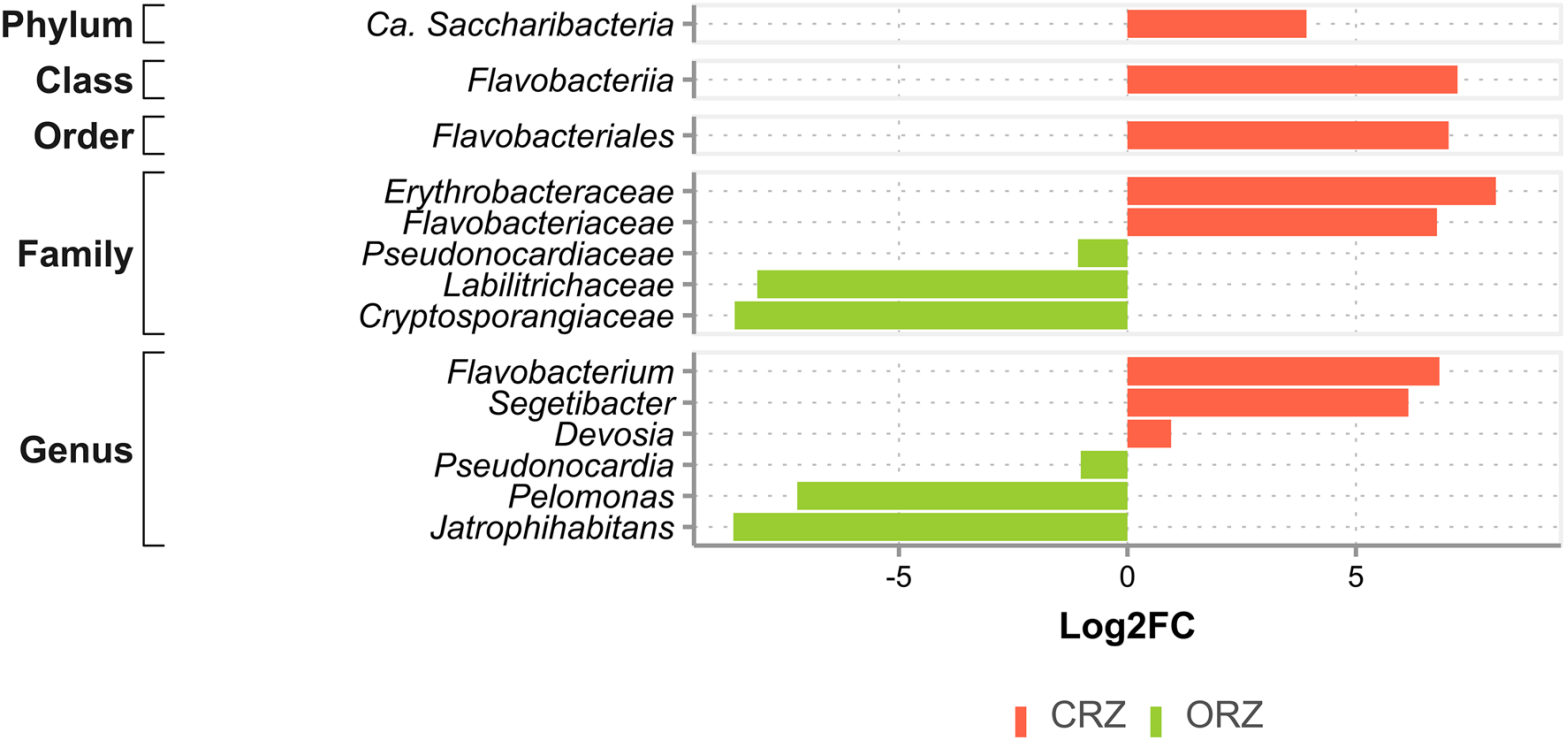
Differently abundant prokaryotic taxa between conventional (CRZ) and organic (ORZ) farming systems present in the sugarcane rhizosphere microbiota, through statistical verification by the Wald test, considering a *p*-value ≤ 0.01.

In total, 14 bacterial taxa were found to be differentially abundant in both farming systems (Fig. 8; Supplementary Table 7). Of these, 8 taxa had significant differences in abundance, considering a *p*-value ≤ 0.01 in a conventional farming system. Among them, are the phylum Saccharibacteria, the class Flavobacteria, and the order Flavobacteriales. Two families are highlighted, *Erythrobacteraceae* and *Flavobacteriaceae*, in addition to three genera, *Flavobacterium*, *Segetibacter,* and, to a lesser extent, *Devosia*.

In the organic system, 6 taxa were differentially abundant in relation to the conventional one, considering a *p*-value ≤ 0.01. Among these, two families are *Labilitrichaceae*, *Cryptosporangiaceae*, and, to a lesser extent, *Pseudonocardiaceae*. The three genera identified, *Jatrophihabitans*, *Pelomonas*, and *Pseudonocardia,* were the most abundant in the organic system, respectively to the referred families.

**Figure 9:**
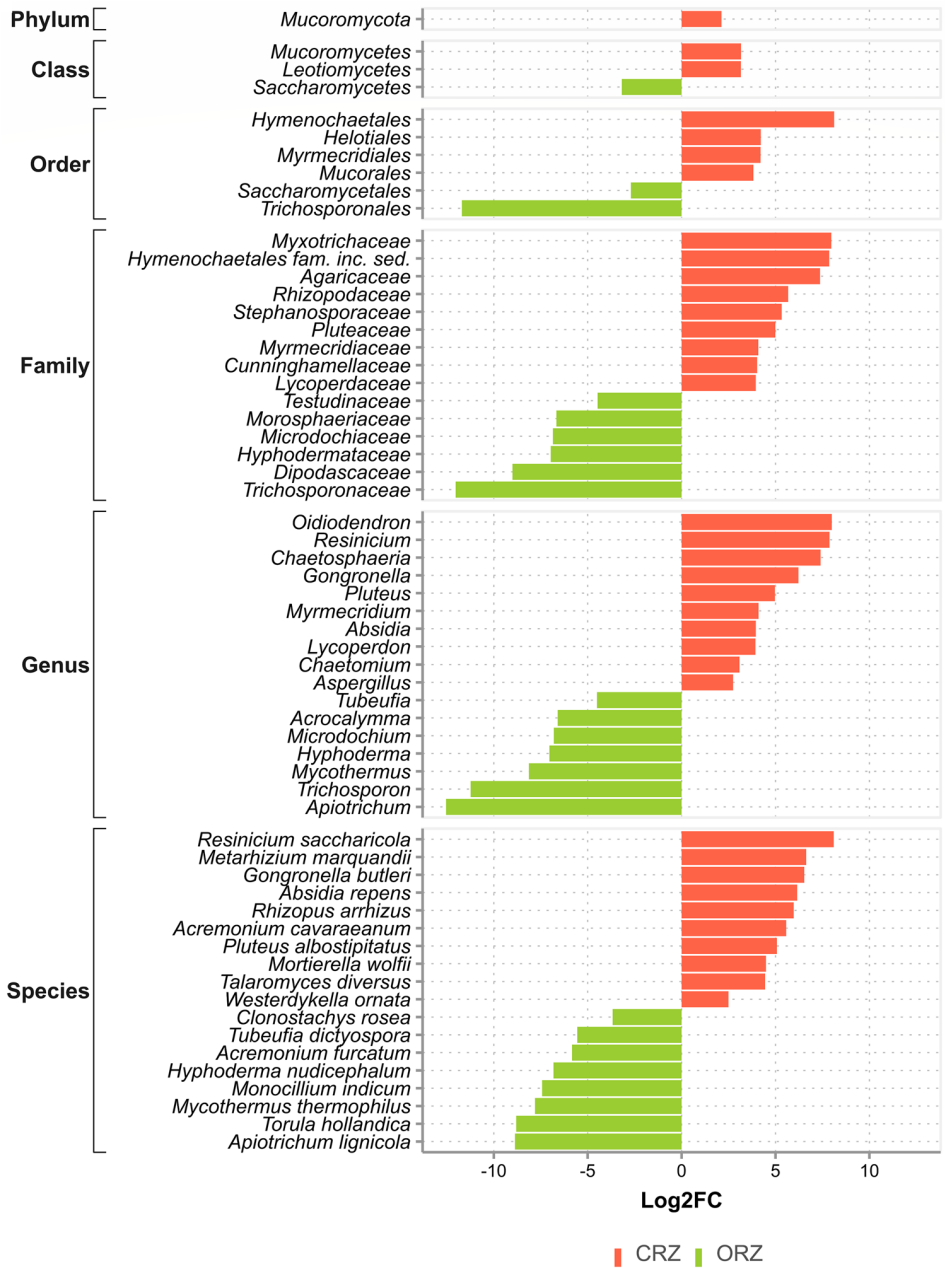
Differently abundant fungal taxa between conventional (CRZ) and organic (ORZ) farming systems present in the sugarcane rhizosphere microbiota, through statistical verification by the Wald test, considering a *p*-value ≤ 0.01.

For fungi, 60 differentially abundant taxa were found, demonstrating greater expressiveness when compared to bacterial taxonomy (Fig. 9; Supplementary Table 8). Of these, 36 taxa were differentially abundant in conventional cultivation and 24 taxa in organic cultivation, considering a p-value ≤ 0.01.

### Predicted functional profiling comparison between the farming systems

**Figure 10:**
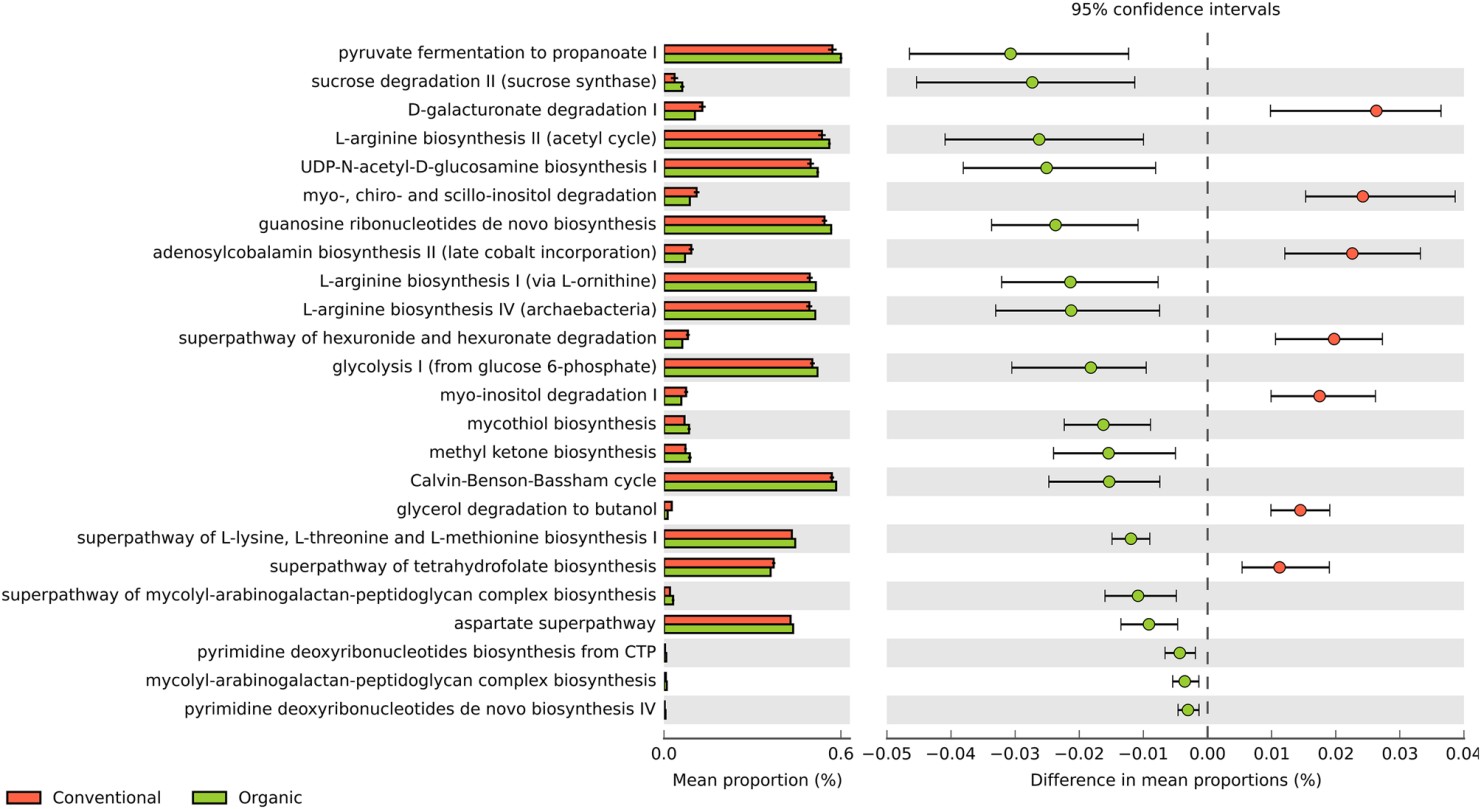
Metabolic pathways of prokaryotes, differentially enriched in the sugarcane rhizosphere in the organic versus conventional farming system. The White’s t-test was used for significance evaluation, considering a *p*-value ≤ 0.05 as statistically significant. The pathways in red represent the conventional system and in green, the organic one.

In addition to the differences observed in the composition and diversity, we evaluate the differences in the predicted functional profiling of microbial communities found in both the sugarcane rhizosphere of the contrasting farming systems. In total, 324 metabolic pathways were identified in the prokaryotic dataset, among which 24 pathways were slightly differentially enriched, considering a *p*-value ≤ 0.05. Of these, 17 were differentially abundant in the organic system and 7 in the conventional one (Fig. 10).

**Figure 11:**
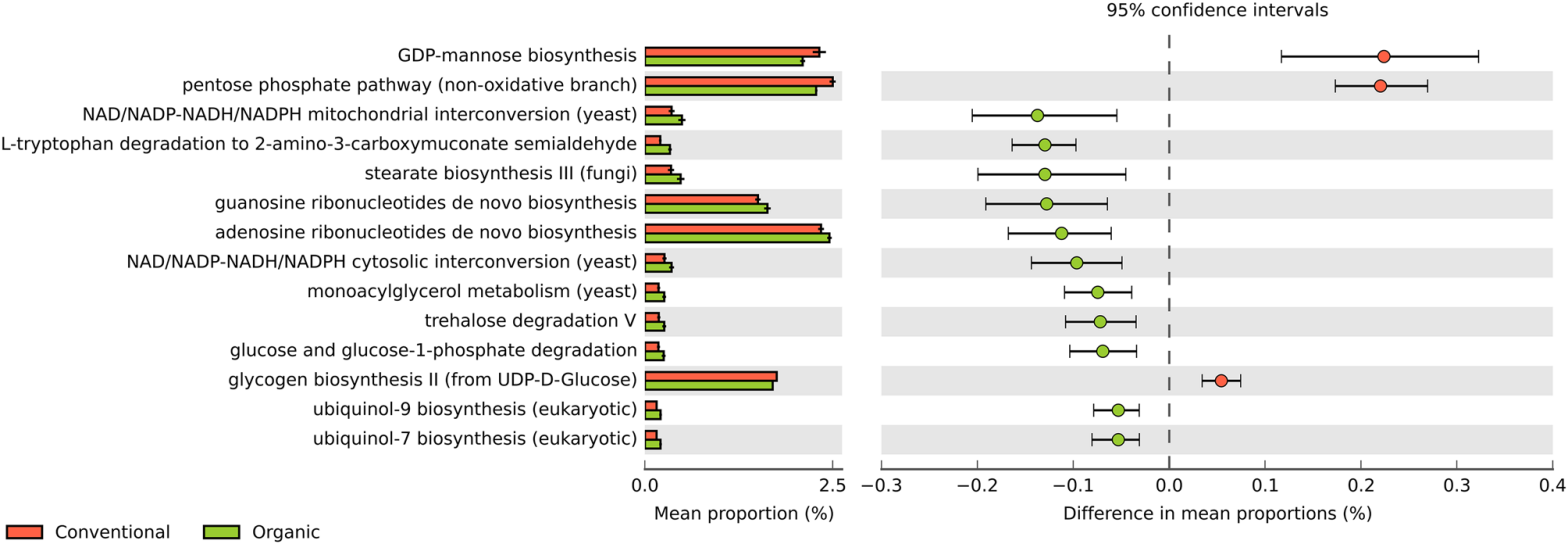
Metabolic pathways of fungi, differentially enriched in the sugarcane rhizosphere in the organic versus conventional farming system. The White’s t-test was used for significance tests, considering a *p*-value ≤ 0.05 as statistically significant. The pathways in red represent the conventional system and in green, the organic one.

Regarding the predicted functional profiling of fungal communities, 69 metabolic pathways were identified, of which 14 were more prominent within the analyzed samples, considering a *p*-value ≤ 0.05. Of these, 11 had higher proportions in the organic system and only 3 in the conventional one (Fig. 11).

### Structures of the microbial co-occurrence networks

**Figure 12:**
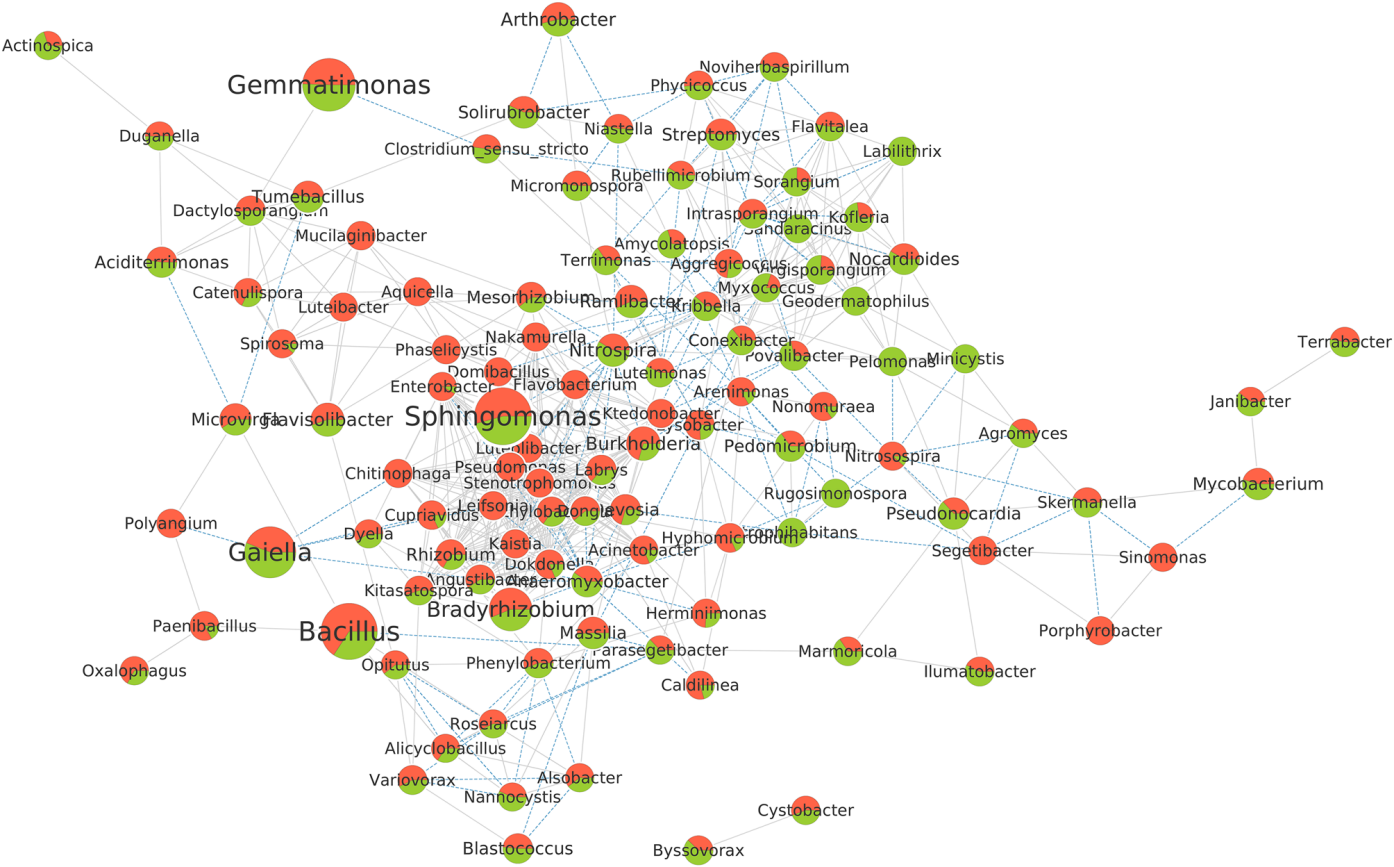
Co-occurrence network of prokaryotic genera present in conventional and organic farming systems. The circles are proportional to the sums of the relative abundance of each genus. The color indicates the relative abundance in the conventional (red) and organic (green) systems. Gray lines are indicative of positively correlated connections between genera, while blue lines are negatively correlated connections. The measures of centrality of the treatments were statistically compared, using the parameters of minimum correlation =  ± 0.5 and *p*-value ≤ 0.05.

To investigate the structures of microbial communities, co-occurrence networks of genera identified in the prokaryotic and fungal datasets were performed. Among all the prokaryotic genera, 110 passed the filtering criteria (minimum correlation coefficient and significance) and were considered for the build of the co-occurrence networks for the conventional and organic farming systems (Fig. 12). The genus *Burkholderia* stands out with greater connectivity with other bacterial genera, totaling 25 correlations (Supplementary Table 9). The genera *Nitrosospira*, *Ktedonobacter,* and *Kribbella* had the highest values for betweenness centrality and could be considered key taxa for the prokaryotic network (Supplementary Table 10).

**Figure 13:**
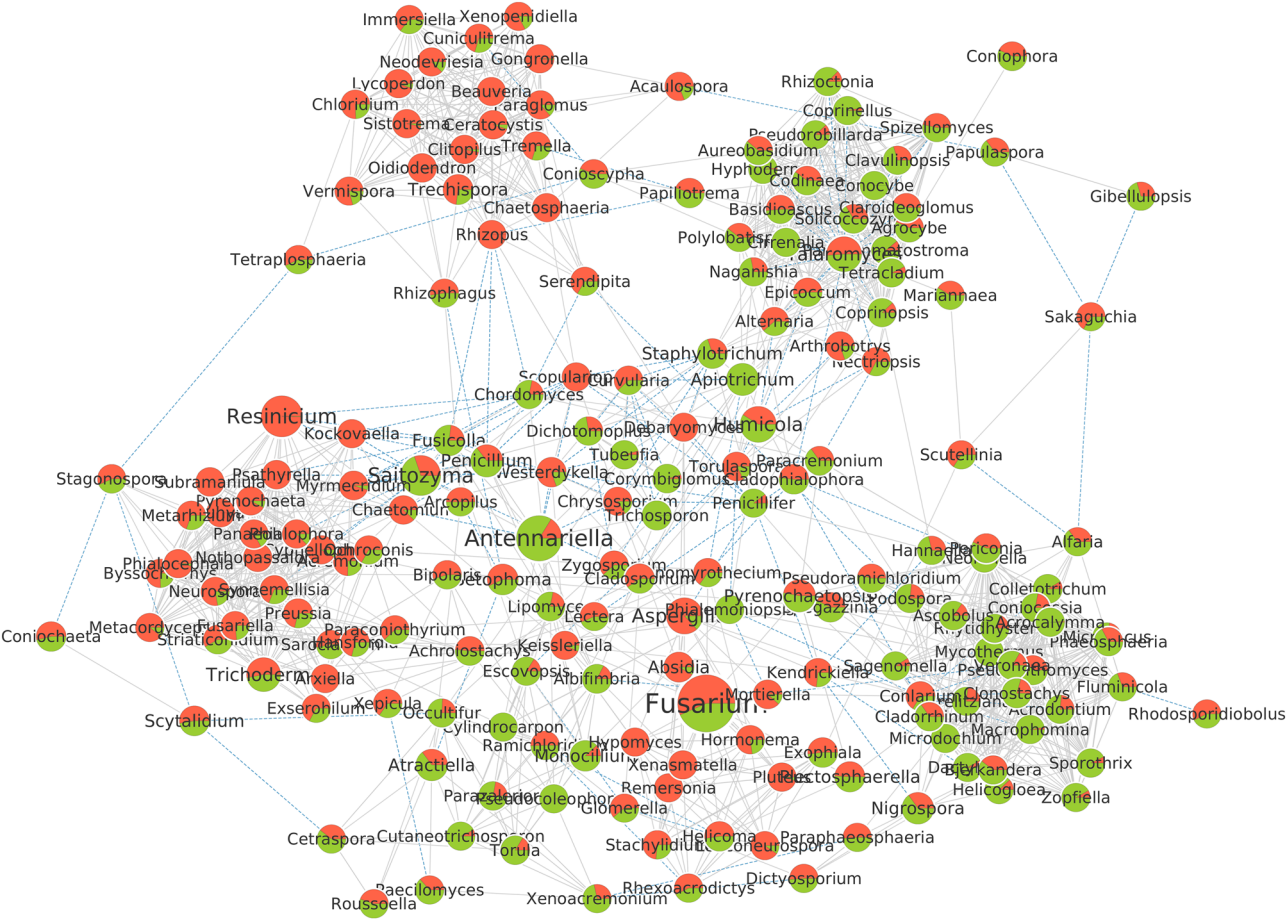
Co-occurrence network of fungal genera present in conventional and organic farming systems. The circles are proportional to the sums of the relative abundance of each genus. The color indicates the relative abundance in the conventional (red) and organic (green) systems. Gray lines are indicative of positively correlated connections between genera, while blue lines are negatively correlated connections. The measures of centrality of the treatments were statistically compared, using the parameters of minimum correlation =  ± 0.5 and *p*-value ≤ 0.05.

Regarding the fungal dataset, 187 taxa passed the filtering criteria and were considered for the build of the fungal co-occurrence network, with *Fusarium* being the most abundant genus present in the farming systems (Fig. 13). The genus *Ascobolus* was more connected to other fungal genera, totaling 27 connections (Supplementary Table 11). The genera *Rhizopus*, *Scopulariopsis*, *Podospora*, and *Cladophialophora* had the highest values for betweenness centrality, and suggestively stand out as key taxa in this network (Supplementary Table 12).

## Discussion

The main purpose of this study was to carry out a comparative and investigative analysis of microbial communities present in the sugarcane rhizosphere, under contrasting farming systems, in order to understand the ecological dynamics in terms of composition and diversity. It is possible to note that the physical and chemical parameters of the soil have a great similarity between crops, with a small but statistically significant difference in the acidity parameters (Supplementary Table 4). However, such differences may have not been enough to cause a great impact on the large-scale composition (Figs. 1 and 2) and microbial diversity between the systems (Fig. 3), despite the soil pH may have a strong correlation with microbial diversity, the general composition of the community or for the relative abundance of individual taxonomic groups^51–53^. The slightly higher acidity in the conventional soil was possibly caused by the application of agrochemical inputs and intensive use of nitrogen fertilization^54^. It is also recognized that long-term consecutive cultivation of sugarcane leads to a decline in soil pH and can cause considerable changes in the composition and function of the microbiota^26^.

The alpha diversity indices did not show significant differences between farming systems (considering a *p*-value ≤ 0.1), except in the case of fungal diversity provided by the Gini-Simpson index, which was higher in the conventional system (Fig. 3). For the beta diversity indices, the multivariate permutational analysis of variance (PERMANOVA) showed that there are significant compositional differences (considering a *p*-value ≤ 0.1) when evaluating only the treatment (conventional or organic) as a descriptive factor of heterogeneity between both systems (Figs. 4 and 5). The results show that the effects arising from these farming systems occur in specific microbial taxa, and this does not have a systemic impact on the diversity of the entire microbial community, where variations in certain groups can be counterbalanced by opposite variations in others^55^. For sugarcane, genotype influences on the modulation of the associated microbiota are reported^17,18^. Even so, these results are according to previous studies which placed the soil properties (i.e., soil texture, water content, and soil type) and host plant (i.e., plant species) as the main drivers of the rhizosphere microbiome assembly^56^, since the soils were very similar and the cultivar was the same in both systems.

The higher fungal diversity in conventional cultivation suggests the potential of a plant to select fungal communities in the rhizospheric environment through the composition of its root exudates^57^. The collections of rhizospheric material in each crop were carried out at the end of the vegetative growth period (11 months) and the beginning of the maturation of the sugarcane field, and it can be inferred that different stages of plant growth can determine its composition, amount of rhizodeposits present and its associated microbiome^57,58^. During plant growth, many ecological succession processes can occur, resulting in new habitats and an increase in the breadth of niches^59^, in our study, we characterized the microbiota only at one specific time point.

As seen in a previous study by de Souza et al.^16^, core microorganisms in sugarcane, regardless of fertilization, can bring benefits and plant vitality. In our study, we identified members of the sugarcane rhizosphere core microbiome (Figs. 6 and 7). Notably, among bacterial genera, *Sphingomonas*, *Gemmatimonas*, *Gaiella*, *Bacillus*, and *Bradyrhizobium*, and among the fungal genera, *Trichoderma* stand out as potential plant growth promoters, phytopathogen inhibitors and participants in soil nutrient cycling^51,60–64^. The same was observed for *Antennariella placitae* and *Saitozyma podzolica*, underreported fungal species that demonstrate potential for biological control in rice and apple plants^65,66^.

The microbial community structure, even in environments with climatic and soil type similarities, may differ according to the agricultural practice employed, selecting specific microorganisms^67^. In the conventional system, differentially abundant bacteria were found (Fig. 8), such as those of the *Flavobacterium* genus, which can perform heterotrophic denitrification^68^ and degradation of various pesticides^69–71^. The *Devosia* genus, also found differently abundant in the conventional system (Fig. 8), was often found in environments contaminated with hydrocarbon pesticides and hexachlorocyclohexane, considered a biodetoxification agent^72,73^.

The increased abundance of specific microbial taxa, possibly caused by long-term fertilization, may demonstrate a direct link with soil nutrients^74^, as seen in organic farming (Fig. 8). In it, the differentially abundant bacterial phyla were classified as Actinobacteria and Betaproteobacteria, which ecologically have a copiotrophic life strategy, with rapid growth in soils with high nutritional availability^54,75^. It has been reported that microbial communities associated with organic management practices tend to be copiotrophic, due to high concentration of nutrients, greater availability and utilization of nitrogen and organic carbon^76,77^. Organic soils are major sources of recalcitrant carbon^78^, which explains the high prevalence of Actinobacteria in this system, confirming the importance of this taxon in the carbon cycle and for the decomposition of this element^79,80^. This confirms that classifications at high taxonomic levels can predictably respond to environmental variables, resulting in high ecological coherence^74^. Ecological coherence demonstrates that the abundance of certain bacterial phyla can change directionally to the type of long-term fertilization employed^74^.

In the organic system (Fig. 9), members of the phylum Ascomycota are predominant. Most saprotrophic microfungi falls under this phylum, and stands out for their importance in the decomposition of organic substrates^81,82^. Also, it is acknowledged that Ascomycota is positively associated with organic matter and nitrogen present in the sugarcane soil^83^. Although differences in these elements are not noticed in our study, it could be an ongoing shift related to the application of organic inputs during the planting period in this system. In the conventional system (Fig. 9), the fungal phyla were more heterogeneous, contrary to what was reported by Lupatini et al.^76^. As seen by Paungfoo-Lonhienne et al.^82^, the use of NPK fertilizer has been associated with the presence and increase of fungal biomass in the sugarcane rhizosphere, which may lead to changes in the composition of fungal communities. Long-term application of mineral fertilizers provides large amounts of nutrients to the soil. These introduced nutrients can increase exudation and alter the fungal community present^57^. This is because fertilization directly influences the physiological state of the plant and favors the release of these exudates^84^. The use of inorganic nitrogen can reduce the dependence of rhizosphere communities on the use of plant-derived carbon and activate many dormant fungal species^57^. This applies to the fact that most fungi are heterotrophs and highly dependent on exogenous carbon for their growth^85^. The release of root exudates may gradually decrease or cease as the plant matures and reaches senescence and the microorganisms obtain their nutrients from the soil^84^.

The functions and metabolic pathways associated with the rhizosphere microbiota from both systems were predicted and evaluated using an enrichment analysis (Figs. 10 and 11). Although there were a few shifts between the systems, it was not possible to identify notable associations with the influences caused by the type of agricultural management, since the vast majority of enrichments were related to the structural and biological processes of the microbiomes. Despite inoculation with nitrogen-fixing bacteria in the organic system (Supplementary Table 2), pathways related to this process were not affected by treatments. Nonetheless, it is worth mentioning that the process of biological nitrogen fixation catalyzed by nitrogenases is dependent on the micronutrients Iron (Fe), Vanadium (V), and, mainly, Molybdenum (Mo)^86^, which has not been evaluated in the present study.

As seen by Schmidt et al.^87^, the type of management employed can determine the microbial community structure, i.e. the taxa and their interactions in a co-occurrence network, leading to important ecological and agricultural inferences. The analyzes of bacterial and fungal co-occurrence networks in organic and conventional farming systems demonstrate distinct patterns of connections, through different microbial identities and abundances, even though these crops share similar climate and soil conditions (Figs. 12 and 13). These microbial networks comprise parasitic, amensalistic, commensalistic, synergistic, or mutualistic interactions that influence each of their constituents and may produce effects on plant health and soil fertility^67^. Our results suggest, through high values ​​of betweenness centrality, the presence of key interconnected taxa in the network that are highly important for the formation of microbial communities in their host plants, controlling or inhibiting the colonization by other microorganisms^88^.

Both systems have highly connected key taxa with different identities, demonstrating that these crops have important taxa that vary considerably^67^. Betweenness centrality is usually described as an indication of key taxa, although this metric can be confirmed only through experimental validation^87^. The highest intermediation centrality in the bacterial network was represented by the genus *Nitrosospira*, a well-recognized ammonia oxidant, present in high abundance in conventional cultivation^89^. A great abundance of *Nitrosospira* in soils that receive nitrogen fertilization has been reported, which may lead to a significant increase in the process of soil nitrification compared to organic treatment^90^. This specialized metabolic function present in *Nitrosospira* may be critical to the stability of the soil microbiome^91^. In the fungal network, *Rhizopus* (Mucoromycota) present exclusively in conventional cultivation was considered a key taxon, with the highest betweenness centrality value. This genus may have had its abundance favored in a conventional system by the nitrogen fertilization used, which suggestively led to an increase in fungal diversity in this system. The greater diversity may have been driven by the high nutritional increment and the rhizospheric exudation stimulated by the inorganic nitrogen fertilization used^57,82,84^.

## Conclusions

Through this study, we could identify slight variations in the rhizosphere microbiome of sugarcane plants when comparing organic and conventional farming systems. We could not directly associate the identified variation with the physical and chemical properties of the soil, because we do not find substantial evidence indicating that the organic or conventional farming system influenced these soil properties. It is improbable that the slight observed differences in pH have a direct relationship with the differences in microbial composition and diversity observed between these crops.

The results show that there are some differences in beta diversity related to the systems. However, such differences could not lead to a substantial effect on the alpha diversity and taxonomic composition at phylum and order levels, according to descriptive levels of statistical significance. Despite this, our study allowed us to recognize that the contrasting systems present the presence of differentially abundant taxa when analyzed at more specific levels, presumably caused by the farming systems. With this, we can assume that agricultural practices can subtly influence the rhizosphere microbiota.

The management systems suggestively may have influenced the structure of interactions revealed by the co-occurrence networks of both microbiotas. The rhizosphere involves different types of interactions between microorganisms, through their root exudates that can shape the structure and a large part of the composition and activities of microbial communities. In particular, in the case of fungi, we can clearly observe differences in their structuring due to changes in the abundance of certain genera and increased diversity caused by the conventional cultivation system, which lead to changes in ecological relationships. In addition, the central microbiome of the sugarcane rhizosphere, that is, the microorganisms independent of the adopted cropping system, revealed taxa known as plant growth promoters. We can consider that the understanding of these microbial relationships is fundamental for the development of a more sustainable agriculture.

The great diversification of factors that involve and change the composition and structure of communities, in addition to the type of agricultural practice, leads to the need for deeper analysis. Thus, the types of regimens employed and their effects on the microbial community should be analyzed more comprehensively, using molecular approaches and identifying more precisely the proportion of the typical variations. This means that we cannot rule out the possibility of more expressive differences considering other conditions, for example, other plants, soils, climates, handling, collections, preparations, products used, crop rotation, number of consecutive harvests (cuts), in particular the time of conversion to the organic system. Thus, more research is needed to investigate the impact of each of these factors considering long-term agricultural systems.

## Supplementary Information

Supplementary Information.

## Data Availability

The datasets generated during and/or analysed during the current study are available in the Sequence Read Archive (SRA) of NCBI under the accession number PRJNA873945 (https://www.ncbi.nlm.nih.gov/bioproject/PRJNA873945).

## References

[1] Meghana M, Shastri Y (2020). Sustainable valorization of sugar industry waste: Status, opportunities, and challenges. Biores. Technol..

[2] Petrescu DC, Vermeir I, Petrescu-Mag RM (2019). Consumer understanding of food quality, healthiness, and environmental impact: a cross-national perspective. IJERPH.

[3] Kassam A, Friedrich T, Shaxson F, Pretty J (2009). The spread of conservation agriculture: justification, sustainability and uptake. Int. J. Agric. Sustain..

[4] Malviya MK, Solanki M K, Li C-N, Htun R, Singh RK, Singh P, Li Y-R (2021). Sugarcane microbiome: role in sustainable production. Microbiomes and Plant Health.

[5] Sandhu HS, Wratten SD, Cullen R (2010). Organic agriculture and ecosystem services. Environ. Sci. Policy.

[6] Schipanski ME (2017). Balancing multiple objectives in organic feed and forage cropping systems. Agr. Ecosyst. Environ..

[7] Knapp S, van der Heijden MGA (2018). A global meta-analysis of yield stability in organic and conservation agriculture. Nat. Commun..

[8] Bender SF, Wagg C, van der Heijden MGA (2016). An underground revolution: biodiversity and soil ecological engineering for agricultural sustainability. Trends Ecol. Evol..

[9] Berendsen RL, Pieterse CMJ, Bakker PAHM (2012). The rhizosphere microbiome and plant health. Trends Plant Sci..

[10] Chialva M, Lanfranco L, Bonfante P (2022). The plant microbiota: composition, functions, and engineering. Curr. Opin. Biotechnol..

[11] Dastogeer KMG, Tumpa FH, Sultana A, Akter MA, Chakraborty A (2020). Plant microbiome–an account of the factors that shape community composition and diversity. Curr. Plant Biol..

[12] Yang B, Wang Y, Qian P-Y (2016). Sensitivity and correlation of hypervariable regions in 16S rRNA genes in phylogenetic analysis. BMC Bioinformat..

[13] Nilsson RH (2019). The UNITE database for molecular identification of fungi: handling dark taxa and parallel taxonomic classifications. Nucleic Acids Res..

[14] Wright RJ, Gibson MI, Christie-Oleza JA (2019). Understanding microbial community dynamics to improve optimal microbiome selection. Microbiome.

[15] Praeg N, Illmer P (2020). Microbial community composition in the rhizosphere of *Larix decidua* under different light regimes with additional focus on methane cycling microorganisms. Sci. Rep..

[16] de Souza RSC (2016). Unlocking the bacterial and fungal communities assemblages of sugarcane microbiome. Sci. Rep..

[17] Tayyab M (2022). Sugarcane cultivars manipulate rhizosphere bacterial communities’ structure and composition of agriculturally important keystone taxa. 3 Biotech..

[18] Tayyab M (2022). Sugarcane cultivar-dependent changes in assemblage of soil rhizosphere fungal communities in subtropical ecosystem. Environ. Sci. Pollut. Res..

[19] Dakora FD, Matiru VN, Kanu AS (2015). Rhizosphere ecology of lumichrome and riboflavin, two bacterial signal molecules eliciting developmental changes in plants. Front. Plant Sci..

[20] Chapelle E, Mendes R, Bakker PAH, Raaijmakers JM (2016). Fungal invasion of the rhizosphere microbiome. ISME J..

[21] Teheran-Sierra LG (2021). Bacterial communities associated with sugarcane under different agricultural management exhibit a diversity of plant growth-promoting traits and evidence of synergistic effect. Microbiol. Res..

[22] de Carvalho LAL (2021). Farming systems influence the compositional, structural, and functional characteristics of the sugarcane-associated microbiome. Microbiol. Res..

[23] Henneron L (2015). Fourteen years of evidence for positive effects of conservation agriculture and organic farming on soil life. Agron. Sustain. Dev..

[24] Hartmann M, Frey B, Mayer J, Mäder P, Widmer F (2015). Distinct soil microbial diversity under long-term organic and conventional farming. ISME J..

[25] Tayyab M (2021). Sugarcane monoculture drives microbial community composition, activity and abundance of agricultural-related microorganisms. Environ. Sci. Pollut. Res..

[26] Pang Z (2021). Soil Metagenomics reveals effects of continuous sugarcane cropping on the structure and functional pathway of rhizospheric microbial community. Front. Microbiol..

[27] Orr CH, Stewart CJ, Leifert C, Cooper JM, Cummings SP (2015). Effect of crop management and sample year on abundance of soil bacterial communities in organic and conventional cropping systems. J. Appl. Microbiol..

[28] Brasil. Lei n^o^ 10.831, de 23 de dezembro de 2003. Dispõe sobre a agricultura orgânica e dá outras providências. In *Publicado no Diário Oficial da União de 24/12/2003* (2003).

[29] Europea C (2007). Reglamento (CE) n^o^ 834/2007 del Consejo, de 28 de junio de 2007, sobre producción y etiquetado de los productos ecológicos y por el que se deroga el Reglamento (CEE) n^o^ 2092/91. D. Of. Unión Eur..

[30] Council of the European Union (2007). 889/2008, “Commission Regulation 889/2008/EC of 5 September 2008 laying down detailed rules for the implementation of Council Regulation (EC) No 834/2007 on organic production and labelling of organic products with regard to organic production, labelling and control”. Off. J. Eur. Union (L).

[31] de Andrade, J. C., Cantarella, H. & Quaggio, J. A. Análise química para avaliação da fertilidade de solos tropicais. (2001).

[32] Donagema, G. K., de Campos, D. B., Calderano, S. B., Teixeira, W. G. & Viana, J. M. Manual de métodos de análise de solo. In *Embrapa Solos-Documentos (INFOTECA-E)* (2011).

[33] Kassambara, A. *ggpubr: ‘ggplot2’ Based Publication Ready Plots*. (2020). at <https://CRAN.R-project.org/package=ggpubr>

[34] R Core Team. *R: A Language and Environment for Statistical Computing*. (R Foundation for Statistical Computing, 2020). At <https://www.R-project.org/>

[35] Lundberg DS, Yourstone S, Mieczkowski P, Jones CD, Dangl JL (2013). Practical innovations for high-throughput amplicon sequencing. Nat. Methods.

[36] Fadrosh DW (2014). An improved dual-indexing approach for multiplexed 16S rRNA gene sequencing on the Illumina MiSeq platform. Microbiome.

[37] Renaud G, Stenzel U, Maricic T, Wiebe V, Kelso J (2015). deML: robust demultiplexing of Illumina sequences using a likelihood-based approach. Bioinformatics.

[38] Zhang J, Kobert K, Flouri T, Stamatakis A (2014). PEAR: a fast and accurate Illumina Paired-End reAd mergeR. Bioinformatics.

[39] Edgar RC (2010). Search and clustering orders of magnitude faster than BLAST. Bioinformatics.

[40] Callahan BJ (2016). DADA2: high-resolution sample inference from Illumina amplicon data. Nat. Methods.

[41] Cole JR (2014). Ribosomal database project: data and tools for high throughput rRNA analysis. Nucleic Acids Res..

[42] McMurdie PJ, Holmes S (2013). phyloseq: an R package for reproducible interactive analysis and graphics of microbiome census data. PLoS One.

[43] Lahti, L. & Shetty, S. *Microbiome R package*. (2012).

[44] Oksanen, J. *et al.**vegan: Community Ecology Package*. (2019). At <https://CRAN.R-project.org/package=vegan>

[45] Love MI, Huber W, Anders S (2014). Moderated estimation of fold change and dispersion for RNA-seq data with DESeq2. Genome Biol..

[46] Dhariwal A (2017). MicrobiomeAnalyst: a web-based tool for comprehensive statistical, visual and meta-analysis of microbiome data. Nucleic Acids Res..

[47] Douglas GM (2019). PICRUSt2: an improved and extensible approach for metagenome inference. Bioinformatics.

[48] Parks DH, Tyson GW, Hugenholtz P, Beiko RG (2014). STAMP: statistical analysis of taxonomic and functional profiles. Bioinformatics.

[49] Kohl M, Wiese S, Warscheid B, Hamacher M, Eisenacher M, Stephan C (2011). Cytoscape: software for visualization and analysis of biological networks. Data Mining in Proteomics.

[50] Assenov Y, Ramírez F, Schelhorn S-E, Lengauer T, Albrecht M (2008). Computing topological parameters of biological networks. Bioinformatics.

[51] Shen Z (2014). Deep 16S rRNA pyrosequencing reveals a bacterial community associated with banana fusarium wilt disease suppression induced by bio-organic fertilizer application. PLoS One.

[52] Yun Y (2016). The relationship between pH and bacterial communities in a single karst ecosystem and its implication for soil acidification. Front. Microbiol..

[53] Wu Y, Zeng J, Zhu Q, Zhang Z, Lin X (2017). pH is the primary determinant of the bacterial community structure in agricultural soils impacted by polycyclic aromatic hydrocarbon pollution. Sci. Rep..

[54] Li R (2012). Pyrosequencing reveals the influence of organic and conventional farming systems on bacterial communities. PLoS One.

[55] Bill M, Chidamba L, Gokul JK, Labuschagne N, Korsten L (2021). Bacterial community dynamics and functional profiling of soils from conventional and organic cropping systems. Appl. Soil. Ecol..

[56] Xun W, Shao J, Shen Q, Zhang R (2021). Rhizosphere microbiome: Functional compensatory assembly for plant fitness. Comput. Struct. Biotechnol. J..

[57] Semenov MV, Krasnov GS, Semenov VM, van Bruggen A (2022). Mineral and organic fertilizers distinctly affect fungal communities in the crop rhizosphere. JoF.

[58] Wang Z, Li Y, Li T, Zhao D, Liao Y (2020). Tillage practices with different soil disturbance shape the rhizosphere bacterial community throughout crop growth. Soil Tillage Res..

[59] Gdanetz K, Trail F (2017). The wheat microbiome under four management strategies, and potential for endophytes in disease protection. Phytobiom. J..

[60] Lazcano C (2021). The rhizosphere microbiome plays a role in the resistance to soil-borne pathogens and nutrient uptake of strawberry cultivars under field conditions. Sci. Rep..

[61] Leys NMEJ (2004). Occurrence and phylogenetic diversity of *Sphingomonas* strains in soils contaminated with polycyclic aromatic hydrocarbons. Appl. Environ. Microbiol..

[62] Yin C (2013). Role of bacterial communities in the natural suppression of rhizoctonia solani bare patch disease of wheat (*Triticum aestivum* L.). Appl. Environ. Microbiol..

[63] Stewart A, Hill R (2014). Applications of trichoderma in plant growth promotion. Biotechnology and Biology of Trichoderma.

[64] Banerjee S (2016). Network analysis reveals functional redundancy and keystone taxa amongst bacterial and fungal communities during organic matter decomposition in an arable soil. Soil Biol. Biochem..

[65] Andargie M, Congyi Z, Yun Y, Li J (2017). Identification and evaluation of potential bio-control fungal endophytes against Ustilagonoidea virens on rice plants. World J. Microbiol. Biotechnol..

[66] Orrù L (2021). How tillage and crop rotation change the distribution pattern of fungi. Front. Microbiol..

[67] van der Heijden MGA, Hartmann M (2016). Networking in the plant microbiome. PLoS Biol..

[68] Wang W (2016). Consistent responses of the microbial community structure to organic farming along the middle and lower reaches of the Yangtze River. Sci. Rep..

[69] Silva TM (2007). Degradation of 2,4-D herbicide by microorganisms isolated from Brazilian contaminated soil. Braz. J. Microbiol..

[70] Laura M, Snchez-Salinas E, Gonzlez ED, Luisa M, Chamy R (2013). Pesticide biodegradation: mechanisms, genetics and strategies to enhance the process. Biodegradation - Life of Science.

[71] Upadhyay LSB, Dutt A, Patra JK, Vishnuprasad CN, Das G (2017). Microbial detoxification of residual organophosphate pesticides in agricultural practices. Microbial Biotechnology.

[72] Hassan YI, Lepp D, He J, Zhou T (2014). Draft genome sequences of *Devosia* sp. strain 17-2-E-8 and Devosia riboflavina strain IFO13584. Genome Announ..

[73] Talwar C (2020). Defining the environmental adaptations of genus Devosia: insights into its expansive short peptide transport system and positively selected genes. Sci. Rep..

[74] Li F, Chen L, Zhang J, Yin J, Huang S (2017). Bacterial community structure after long-term organic and inorganic fertilization reveals important associations between soil nutrients and specific taxa involved in nutrient transformations. Front. Microbiol..

[75] Ho A, Lonardo DPD, Bodelier PLE (2017). Revisiting life strategy concepts in environmental microbial ecology. Microbiol. Ecol..

[76] Lupatini M, Korthals GW, de Hollander M, Janssens TKS, Kuramae EE (2017). Soil microbiome is more heterogeneous in organic than in conventional farming system. Front. Microbiol..

[77] Wang H (2021). Eight years of manure fertilization favor copiotrophic traits in paddy soil microbiomes. Eur. J. Soil Biol..

[78] Fließbach A, Oberholzer H-R, Gunst L, Mäder P (2007). Soil organic matter and biological soil quality indicators after 21 years of organic and conventional farming. Agric. Ecosyst. Environ..

[79] Lewin GR (2016). Evolution and ecology of *Actinobacteria* and their bioenergy applications. Annu. Rev. Microbiol..

[80] Karanja EN (2020). Diversity and structure of prokaryotic communities within organic and conventional farming systems in central highlands of Kenya. PLoS One.

[81] Francioli D (2016). Mineral versus organic amendments: microbial community structure, activity and abundance of agriculturally relevant microbes are driven by long-term fertilization strategies. Front. Microbiol..

[82] Paungfoo-Lonhienne C (2015). Nitrogen fertilizer dose alters fungal communities in sugarcane soil and rhizosphere. Sci. Rep..

[83] Pang Z (2019). Liming positively modulates microbial community composition and function of sugarcane fields. Agronomy.

[84] Aira M, Gómez-Brandón M, Lazcano C, Bååth E, Domínguez J (2010). Plant genotype strongly modifies the structure and growth of maize rhizosphere microbial communities. Soil Biol. Biochem..

[85] Ma M (2018). Responses of fungal community composition to long-term chemical and organic fertilization strategies in Chinese Mollisols. MicrobiologyOpen.

[86] Bellenger JP, Darnajoux R, Zhang X, Kraepiel AML (2020). Biological nitrogen fixation by alternative nitrogenases in terrestrial ecosystems: a review. Biogeochemistry.

[87] Schmidt JE, Vannette RL, Igwe A, Blundell R, Casteel CL, Gaudin ACM (2019). Effects of agricultural management on rhizosphere microbial structure and function in processing tomato plants. Appl. Environ. Microbiol..

[88] Agler MT (2016). Microbial hub taxa link host and abiotic factors to plant microbiome variation. PLoS Biol..

[89] Lin Y (2018). Nitrosospira cluster 8a plays a predominant role in the nitrification process of a subtropical Ultisol under long-term inorganic and organic fertilization. Appl. Environ. Microbiol..

[90] Chu H (2007). Community structure of ammonia-oxidizing bacteria under long-term application of mineral fertilizer and organic manure in a sandy loam soil. Appl. Environ. Microbiol..

[91] Xun W (2021). Specialized metabolic functions of keystone taxa sustain soil microbiome stability. Microbiome.

