# Supplementary material for: Sugarcane cultivation practices modulate rhizosphere microbial community composition and structure
Source: Sci Rep. 2022 Nov 10;12:19174. doi: 10.1038/s41598-022-23562-6 (PMC9649670; doi:10.1038/s41598-022-23562-6)
Supplement: Supplementary file 1 — Supplementary Information. [file 41598_2022_23562_MOESM1_ESM.docx]

**Supplementary Material**

**Sugarcane cultivation practices modulate rhizosphere microbial community composition and structure**

Ana Paula Corrêa Moneda^a,b^, Lucas Amoroso Lopes de Carvalho^a,b^, Luis Guillermo Teheran-Sierra^a,b^, Michelli Inácio Gonçalves Funnicelli^a,b^, Daniel Guariz Pinheiro^a,b,*^

^a^ Laboratory of Bioinformatics, Department of Agricultural, Livestock and Environmental Biotechnology, São Paulo State University (UNESP), School of Agricultural and Veterinary Sciences, Jaboticabal, SP, Brazil

^b^ Graduate Program in Agricultural and Livestock Microbiology, São Paulo State University (UNESP), School of Agricultural and Veterinary Sciences, Jaboticabal, SP, Brazil

* Corresponding author at: Laboratory of Bioinformatics, Department of Agricultural, Livestock and Environmental Biotechnology, São Paulo State University (UNESP), School of Agricultural and Veterinary Sciences, Jaboticabal 14884-900, SP, Brazil.

E-mail addresses: daniel.pinheiro@unesp.br (D.G. Pinheiro)


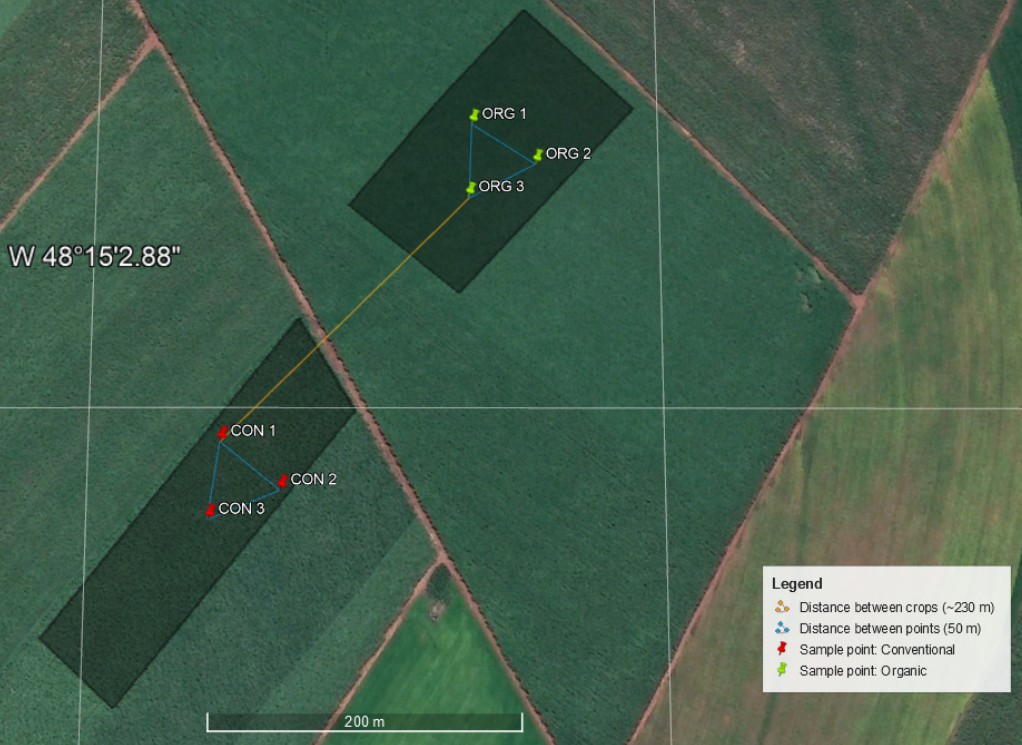


**Supplementary Figure 1.** The sugarcane rhizosphere sampling area for metagenomic DNA extraction at the São José Farm (ORGanic farming system) and at the São Sebastião Farm (CONventional farming system), both in Jaboticabal, São Paulo State, Brazil. The sampling points (right) are equidistant (≈ 50 m; blue line), with the lowest proximity between the sampling points in the Conventional (CON; red pins) and in the Organic (ORG; green pins) cultivation field was approximately 230 m (yellow line).

**Supplementary Table 1.** Geographic coordinates (Latitude and Longitude) and climatological data related to sampling points (Pt.) of sugarcane rhizosphere in the farms under organic (ORG) or conventional (CON) farming systems for the three sampling dates in 2018.

| **Farming System** | **Pt.** | **Latitude** | **Longitude** | **Soil hum. (%)** | **Soil temp. (°C)** | **Atm. temp. (°C)** | **Air hum. (%)** | **Atm. press. (hPa)** | **Date**  **(YYYY-MM-DD)** |
| --- | --- | --- | --- | --- | --- | --- | --- | --- | --- |
| CON | 1 | 21°11'23.27"S | 48°14'59,92"W | 17.98 | 23.0 | 21.42 | 93.01 | 943.70 | 2018-02-27 |
|  | 2 | 21°11'24.28"S | 48°14'58,56"W | 15.70 | 22.9 | 23.49 | 79.94 | 942.50 | 2018-03-03 |
|  | 3 | 21°11'24.88"S | 48°15'00,17"W | 14.84 | 23.5 | 21.20 | 95.24 | 941.00 | 2018-03-08 |
| ORG | 1 | 21°11'16.46"S | 48°14'54,23"W | 17.95 | 22.6 | 25.47 | 75.40 | 944.10 | 2018-02-27 |
|  | 2 | 21°11'17.33"S | 48°14'52,76"W | 17.92 | 23.1 | 28.00 | 61.05 | 943.10 | 2018-03-03 |
|  | 3 | 21°11'18.06"S | 48°14'54,32"W | 17.08 | 22.7 | 24.72 | 78.57 | 941.70 | 2018-03-08 |

Sampling point (Pt.); Relative soil humidity (Soil hum.); Soil temperature (Soil temp.); Atmosphere temperature (Atm. temp.); Relative air humidity (Air hum.); Atmosphere pressure (Atm. press.)

**Supplementary Table 2.** The agricultural inputs used in the organic farming system of sugarcane.

| **Class** | **Product** | **Active ingredient**  **(Chemical Group)** | **Dosage**  **(per ha)** |
| --- | --- | --- | --- |
| Fungicide | Quality® (*) (**) | *Trichoderma asperellum*  1 x 10^10^ CFU/g | 200 g ^(*)^ /100g ^(**)^ |
|  | Difere® | Copper oxychloride 588 g/L | 200 L |
| Nematicide | Rizos® (**) | *Bacillus subtilis*  1,5 x 10^9^ CFU/L | 300 mL ^(**)^ |
|  | Onix® (*) | *Bacillus methylotrophicus* isolado SF 267 1,5  x 10^9^ CFU/L | 0.5 L ^(*)^ |
| Insecticide | Bouveriz ^®^ (*) | *Beauveria bassiana* 8 x 10^9^ | 50 g (*) |
| Plant Growth Promotion | Azos® (*) (**) | *Azospirillum brasilense* estirpe ABV5 1 x 10^9^ CFU/L | 1 L ^(*)^ / 300 mL ^(**)^ |
|  | Starfix® (**) | *Bradyrhizobium japonicum* SEMIA  5079/5080 | 8 doses |

(*) Application at the beginning of planting; (**) Application in the planting furrow of the previous crop

**Supplementary Table 3.** The agrochemical inputs used in the conventional farming system of sugarcane. Percentage of organic/inorganic compounds.

| **Class** | **Product** | **Active ingredient**  **(Chemical group)** | **Dosage**  **(per ha)** |
| --- | --- | --- | --- |
| Insecticide | Orix® (*) | Mineral oil (aliphatic hydrocarbons) | 0.2 L |
|  | Nuprid® | Imidachloropride (Neonicotinoid) | 1 kg |
|  | Regent® (*) | Fipronil (Phenylpyrazole) | 247.93 g |
|  | Engeo Pleno® | Thiamethoxam (Neonicotinoid); Lambda-Cyhalothrin (Pyrethroid) | 1 L |
| Fungicide | Opera® | Epoxiconazole (Triazole); Pyraclostrobin (Strobilurin) | 1 L |
|  | Comet® (*) | Pyraclostrobin (Strobilurin) | 0.495 L |
| Herbicide | Butiron® (*) | Tebuthiuron (Urea) | 1.65 L |
|  | Boral (*) | Sulfentrazone (Triazolone) | 1.65 L |
| Fertilizer | Poliflex® (*) | Phosphorus pentoxide (P2O5) 18%; Nitrogen (N) 3% | 0.062 L |
|  | Zintrac™ (*) | Zinc (Zn) 40%; Nitrogen (N) 1% | 1.033 L |
|  | Bortrac™ (*) | Boron (B) 10,9%; Nitrogen (N) 4,7% | 1.033 L |
|  | Biozyme TF® (*) | Potassium Oxide (K2O) 5,0%; Total Organic Carbon 3,5%; Zinc (Zn) 2%; Nitrogen (N) 1,5%; Manganese (Mn) 1%; Sulfur (S) 1%; Iron (Fe) 0,4%; Boron (B)  0,08% | 0.495 L |

(*) Application at the beginning of planting;

**Supplementary Table 4.** The average values and standard deviation for physicochemical parameters of bulk soil under the organic or conventional farming systems.

| **Soil Parameter** | **Conventional** | **Organic** | **Sig.** |
| --- | --- | --- | --- |
| Organic matter (g.dm^-3^) | 18.92 ± 2.21 | 19.38 ± 1.12 | ns |
| pH | 5.42 ± 0.05 | 5.70 ± 0.25 | ***** |
| potential acidity: H+Al (mmolc.dm^-3^) | 24.32 ± 2.80 | 21.58 ± 3.26 | ***** |
| Sum of Bases (mmolc.dm^-3^) | 38.18 ± 2.78 | 44.61 ± 2.02 | ***** |
| Cation exchange capacity (mmolc.dm^-3^) | 62.50 ± 2.80 | 66.20 ± 3.23 | ns |
| Base saturation (V %) | 61.10 ± 3.97 | 67.47 ± 3.77 | ***** |
| Aluminum saturation (m %) | 0.81 ± 0.76 | 0.15 ± 0.13 | ***** |
| Al (mmolc.dm^-3^) | 0.30 ± 0.26 | 0.07 ± 0.06 | ns |
| B (mg.dm^-3^) | 0.36 ± 0.02 | 0.33 ± 0.03 | ns |
| Ca (mmolc.dm^-3^) | 28.15 ± 3.90 | 32.17 ± 1.39 | ns |
| Cu (mg.dm^-3^) | 3.29 ± 0.80 | 2.91 ± 0.23 | ns |
| Fe (mg.dm^-3^) | 17.18 ± 3.49 | 22.49 ± 3.58 | ns |
| K (mmolc.dm^-3^) | 1.11 ± 0.27 | 1.73 ± 0.41 | ***** |
| Mg (mmolc.dm^-3^) | 8.92 ± 1.43 | 10.72 ± 0.59 | ns |
| Mn (mg.dm^-3^) | 10.43 ± 2.04 | 9.09 ± 1.43 | ns |
| P (mg.dm^-3^) | 13.71 ± 2.18 | 12.78 ± 7.55 | ns |
| S (mg.dm^-3^) | 11.07 ± 7.12 | 5.46 ± 0.36 | ***** |
| Zn (mg.dm^-3^) | 3.03 ± 0.82 | 2.13 ± 0.37 | ns |
| Clay (%) | 32.07 ± 4.02 | 34.99 ± 0.01 | ns |
| Silt (%) | 4.07 ± 1.13 | 6.03 ± 1.33 | ns |
| Sand (%) | 63.86 ± 3.22 | 58.98 ± 1.31 | ***** |
| Coarse sand (%) | 28.01 ± 2.44 | 24.11 ± 1.75 | ns |
| Fine sand (%) | 35.85 ± 1.31 | 34.87 ± 0.45 | ns |

Statistical significance (Sig.) * (p ≤0, 1) and ns (not significant p > 0.1).

**Supplementary Table 5**. High-throughput sequencing and processing results for 16S and ITS amplicons of sugarcane rhizosphere.

| **Dataset** | **Farming**  **system** | **Sample** | **Raw** | **Merged**  **pairs** | **Processed sequences** | **ASVs** | **Assigned at Genus** |
| --- | --- | --- | --- | --- | --- | --- | --- |
| 16S | Organic | ORZ1 | 30,687 | 30,255 | 28,272 | 19,135 | 6,135 |
|  |  | ORZ2 | 51,875 | 51,300 | 48,310 | 36,179 | 11,594 |
|  |  | ORZ3 | 59,385 | 58,805 | 55,229 | 42,251 | 14,668 |
|  | Conventional | CRZ1 | 66,509 | 65,862 | 61,628 | 45,283 | 16,750 |
|  |  | CRZ2 | 66,017 | 65,383 | 61,459 | 46,707 | 18,156 |
|  |  | CRZ3 | 55,212 | 54,669 | 51,405 | 38,052 | 13,162 |
|  | Total |  | 329,685 | 326,274 | 306,303 | 227,607 | 80,465 |
| ITS | Organic | ORZ1 | 47,596 | 47,346 | 43,567 | 42,455 | 27,794 |
|  |  | ORZ2 | 11,676 | 11,441 | 10,426 | 10,042 | 6,329 |
|  |  | ORZ3 | 50,777 | 50,558 | 46,658 | 45,351 | 32,112 |
|  | Conventional | CRZ1 | 66,655 | 66,271 | 60,813 | 58,166 | 32,896 |
|  |  | CRZ2 | 62,383 | 62,073 | 57,374 | 55,932 | 38,595 |
|  |  | CRZ3 | 72,182 | 71,850 | 66,391 | 64,123 | 45,979 |
|  | Total |  | 311,269 | 309,539 | 285,229 | 276,069 | 183,705 |

**Supplementary Table 6.** Alpha diversity indexes’ values of microbial fractions present in three samples of sugarcane rhizosphere under the organic (ORZ) and the conventional (CRZ) farming systems.

|  | **Index** | **Conventional**  **sample** | **Value** | **Organic**  **sample** | **Value** | **Sig.** |
| --- | --- | --- | --- | --- | --- | --- |
| **16S** | **Chao1** | CRZ1 | 370 | ORZ1 | 707 | ns |
|  |  | CRZ2 | 744 | ORZ2 | 784 |  |
|  |  | CRZ3 | 817 | ORZ3 | 739 |  |
|  | **Shannon** | CRZ1 | 5.58 | ORZ1 | 6.28 | ns |
|  |  | CRZ2 | 6.27 | ORZ2 | 6.05 |  |
|  |  | CRZ3 | 6.27 | ORZ3 | 6.28 |  |
|  | **Gini Simpson** | CRZ1 | 0.99 | ORZ1 | 0.99 | ns |
|  |  | CRZ2 | 0.99 | ORZ2 | 0.99 |  |
|  |  | CRZ3 | 0.99 | ORZ3 | 0.99 |  |
| **ITS** | **Chao1** | CRZ1 | 765.23 | ORZ1 | 861.75 | ns |
|  |  | CRZ2 | 670.78 | ORZ2 | 730.59 |  |
|  |  | CRZ3 | 764.7 | ORZ3 | 812.94 |  |
|  | **Shannon** | CRZ1 | 4.92 | ORZ1 | 4.35 | ns |
|  |  | CRZ2 | 4.99 | ORZ2 | 4.59 |  |
|  |  | CRZ3 | 4.66 | ORZ3 | 4.81 |  |
|  | **Gini- Simpson** | CRZ1 | 0.98 | ORZ1 | 0.93 | * |
|  |  | CRZ2 | 0.98 | ORZ2 | 0.97 |  |
|  |  | CRZ3 | 0.97 | ORZ3 | 0.97 |  |

Statistical significance (Sig.) * (p ≤0, 1) and ns (not significant p > 0.1).

**Supplementary Table 7.** Differently abundant prokaryotic taxa obtained using the “DESeq2” approach. Only taxa with statistically significant findings (p-value ≤ 0.01) using the Wald test were considered. The logFc represents the number of times that a specific taxon is in greater abundance in a crop, where negative values indicate greater abundance in organic cultivation and positive values in conventional cultivation.

| Taxon | Taxonomic level | logFC | pvalue | Sample |
| --- | --- | --- | --- | --- |
| *Ca. Saccharibacteria* | Phylum | 3.917 | < 0.001 | CRZ |
| *Flavobacteriia* | Class | 7.221 | < 0.001 | CRZ |
| *Flavobacteriales* | Order | 7.027 | 0.002 | CRZ |
| *Cryptosporangiaceae* | Family | -8.593 | < 0.001 | ORZ |
| *Erythrobacteraceae* | Family | 8.060 | < 0.001 | CRZ |
| *Pseudonocardiaceae* | Family | -1.082 | < 0.001 | ORZ |
| *Flavobacteriaceae* | Family | 6.772 | 0.001 | CRZ |
| *Labilitrichaceae* | Family | - 8.099 | < 0.001 | ORZ |
| *Jatrophihabitans* | Genus | - 8.622 | < 0.001 | ORZ |
| *Pelomonas* | Genus | -7.224 | 0.001 | ORZ |
| *Flavobacterium* | Genus | 6.827 | 0.002 | CRZ |
| *Devosia* | Genus | 0.954 | 0.004 | CRZ |
| *Pseudonocardia* | Genus | -1.020 | 0.006 | ORZ |
| *Segetibacter* | Genus | 6.146 | 0.008 | CRZ |

**Supplementary Table 8:** Differently abundant fungal taxa obtained using the “DESeq2” approach. Only taxa with statistically significant findings (p-value ≤ 0.01) using the Wald test were considered. The logFc represents the number of times that a specific taxon is in greater abundance in a crop, where negative values indicate greater abundance in organic cultivation and positive values in conventional cultivation.

| Taxon | Taxonomic level | logFC | pvalue | Sample |
| --- | --- | --- | --- | --- |
| *Mucoromycota* | Phylum | 2.124 | 0.008 | CRZ |
| *Saccharomycetes* | Class | -3.184 | < 0.001 | ORZ |
| *Mucoromycetes* | Class | 3.162 | 0.004 | CRZ |
| *Leotiomycetes* | Class | 3.155 | 0.004 | CRZ |
| *Trichosporonales* | Order | -11.694 | < 0.001 | ORZ |
| *Hymenochaetales* | Order | 8.122 | < 0.001 | CRZ |
| *Helotiales* | Order | 4.213 | < 0.001 | CRZ |
| *Mucorales* | Order | 3.822 | < 0.001 | CRZ |
| *Myrmecridiales* | Order | 4.200 | 0.001 | CRZ |
| *Saccharomycetales* | Order | -2.696 | 0.005 | ORZ |
| *Trichosporonaceae* | Family | -12.031 | < 0.001 | ORZ |
| *Hymenochaetales* | Family | 7.858 | < 0.001 | CRZ |
| *Dipodascaceae* | Family | -9.005 | < 0.001 | ORZ |
| *Testudinaceae* | Family | -4.471 | < 0.001 | ORZ |
| *Agaricaceae* | Family | 7.371 | < 0.001 | CRZ |
| *Myxotrichaceae* | Family | 7.971 | < 0.001 | CRZ |
| *Hyphodermataceae* | Family | -6.963 | < 0.001 | ORZ |
| *Cunninghamellaceae* | Family | 4.020 | < 0.001 | CRZ |
| *Myrmecridiaceae* | Family | 4.083 | 0.001 | CRZ |
| *Microdochiaceae* | Family | -6.851 | 0.002 | ORZ |
| *Morosphaeriaceae* | Family | -6.666 | 0.003 | ORZ |
| *Pluteaceae* | Family | 4.99 | 0.003 | CRZ |
| *Stephanosporaceae* | Family | 5.326 | 0.004 | CRZ |
| *Lycoperdaceae* | Family | 3.947 | 0.007 | CRZ |
| *Rhizopodaceae* | Family | 5.672 | 0.009 | CRZ |
| *Apiotrichum* | Genus | -12.539 | < 0.001 | ORZ |
| *Trichosporon* | Genus | -11.231 | < 0.001 | ORZ |
| *Resinicium* | Genus | 7.873 | < 0.001 | CRZ |
| *Chaetosphaeria* | Genus | 7.398 | < 0.001 | CRZ |
| *Oidiodendron* | Genus | 7.997 | < 0.001 | CRZ |
| *Hyphoderma* | Genus | -7.037 | < 0.001 | ORZ |
| *Absidia* | Genus | 3.951 | < 0.001 | CRZ |
| *Mycothermus* | Genus | -8.128 | < 0.001 | ORZ |
| *Gongronella* | Genus | 6.219 | 0.001 | CRZ |
| *Tubeufia* | Genus | -4.502 | 0.001 | ORZ |
| *Myrmecridium* | Genus | 4.094 | 0.002 | CRZ |
| *Microdochium* | Genus | -6.804 | 0.002 | ORZ |
| *Acrocalymma* | Genus | -6.596 | 0.003 | ORZ |
| *Pluteus* | Genus | 4.968 | 0.004 | CRZ |
| *Aspergillus* | Genus | 2.740 | 0.008 | CRZ |
| *Chaetomium* | Genus | 3.082 | 0.008 | CRZ |
| *Lycoperdon* | Genus | 3.930 | 0.009 | CRZ |
| *Resinicium saccharicola* | Species | 8.102 | < 0.001 | CRZ |
| *Monocillium indicum* | Species | -7.426 | < 0.001 | ORZ |
| *Absidia repens* | Species | 6.154 | < 0.001 | CRZ |
| *Acremonium cavaraeanum* | Species | 5.565 | < 0.001 | CRZ |
| *Torula hollandica* | Species | -8.805 | < 0.001 | ORZ |
| *Apiotrichum lignicola* | Species | -8.872 | < 0.001 | ORZ |
| *Gongronella butleri* | Species | 6.532 | < 0.001 | CRZ |
| *Talaromyces diversus* | Species | 4.446 | < 0.001 | CRZ |
| *Hyphoderma nudicephalum* | Species | -6.820 | < 0.001 | ORZ |
| *Metarhizium marquandii* | Species | 6.634 | 0.002 | CRZ |
| *Westerdykella ornata* | Species | 2.494 | 0.002 | CRZ |
| *Mycothermus thermophilus* | Species | -7.805 | 0.002 | ORZ |
| *Tubeufia dictyospora* | Species | -5.553 | 0.002 | ORZ |
| *Pluteus albostipitatus* | Species | 5.070 | 0.003 | CRZ |
| *Rhizopus arrhizus* | Species | 5.968 | 0.004 | CRZ |
| *Mortierella wolfii* | Species | 4.488 | 0.008 | CRZ |
| *Clonostachys rosea* | Species | -3.670 | 0.008 | ORZ |
| *Acremonium furcatum* | Species | -5.831 | 0.009 | ORZ |

**Supplementary Table 9:** Prokaryotic genera identified through Pearson's correlation and used as input to the Cytoscape program where, in addition to visual representations, the numbers of correlations (positive and negative) established between the genera were obtained.

| **Taxon** | **Corr.** | **Taxon** | **Corr.** | **Taxon** | **Corr.** |
| --- | --- | --- | --- | --- | --- |
| *Burkholderia* | 25 | *Phaselicystis* | 11 | *Luteimonas* | 6 |
| *Pseudomonas* | 25 | *Rubellimicrobium* | 11 | *Agromyces* | 6 |
| *Methylobacterium* | 24 | *Phenylobacterium* | 10 | *Noviherbaspirillum* | 6 |
| *Stenotrophomonas* | 24 | *Conexibacter* | 10 | *Bacillus* | 5 |
| *Labrys* | 24 | *Pelomonas* | 10 | *Solirubrobacter* | 5 |
| *Sphingomonas* | 24 | *Kofleria* | 10 | *Gaiella* | 5 |
| *Luteolibacter* | 24 | *Nitrosospira* | 9 | *Pedomicrobium* | 5 |
| *Kaistia* | 23 | *Dactylosporangium* | 9 | *Herminiimonas* | 5 |
| *Rhizobium* | 23 | *Arenimonas* | 9 | *Aciditerrimonas* | 5 |
| *Dokdonella* | 22 | *Hyphomicrobium* | 9 | *Nonomuraea* | 5 |
| *Devosia* | 21 | *Phycicoccus* | 9 | *Microvirga* | 4 |
| *Leifsonia* | 21 | *Roseiarcus* | 9 | *Duganella* | 4 |
| *Angustibacter* | 21 | *Streptomyces* | 9 | *Amycolatopsis* | 4 |
| *Bradyrhizobium* | 21 | *Virgisporangium* | 9 | *Micromonospora* | 4 |
| *Enterobacter* | 20 | *Jatrophihabitans* | 8 | *Sinomonas* | 4 |
| *Nakamurella* | 19 | *Catenulispora* | 8 | *Rugosimonospora* | 4 |
| *Kitasatospora* | 19 | *Mucilaginibacter* | 8 | *Dyella* | 4 |
| *Acinetobacter* | 19 | *Luteibacter* | 8 | *Caldilinea* | 4 |
| *Cupriavidus* | 19 | *Labilithrix* | 8 | *Mycobacterium* | 3 |
| *Flavobacterium* | 18 | *Skermanella* | 7 | *Marmoricola* | 3 |
| *Domibacillus* | 18 | *Pseudonocardia* | 7 | *Polyangium* | 3 |
| *Ktedonobacter* | 16 | *Segetibacter* | 7 | *Paenibacillus* | 3 |
| *Sandaracinus* | 16 | *Massilia* | 7 | *Clostridium_sensu_stricto* | 3 |
| *Anaeromyxobacter* | 16 | *Lysobacter* | 7 | *Ramlibacter* | 3 |
| *Dongia* | 16 | *Flavisolibacter* | 7 | *Blastococcus* | 3 |
| *Chitinophaga* | 16 | *Aggregicoccus* | 7 | *Arthrobacter* | 3 |
| *Kribbella* | 15 | *Alicyclobacillus* | 7 | *Porphyrobacter* | 3 |
| *Nitrospira* | 15 | *Minicystis* | 7 | *Janibacter* | 2 |
| *Povalibacter* | 14 | *Alsobacter* | 7 | *Gemmatimonas* | 2 |
| *Sorangium* | 14 | *Nannocystis* | 7 | *Ilumatobacter* | 2 |
| *Nocardioides* | 14 | *Spirosoma* | 7 | *Oxalophagus* | 1 |
| *Intrasporangium* | 13 | *Parasegetibacter* | 6 | *Terrabacter* | 1 |
| *Myxococcus* | 12 | *Tumebacillus* | 6 | *Cystobacter* | 1 |
| *Geodermatophilus* | 12 | *Niastella* | 6 | *Byssovorax* | 1 |
| *Flavitalea* | 12 | *Opitutus* | 6 | *Actinospica* | 1 |
| *Aquicella* | 11 | *Terrimonas* | 6 | *-* | - |
| *Mesorhizobium* | 11 | *Variovorax* | 6 | *-* | - |

**Supplementary Table 10:** Prokaryotic genera identified through Pearson's correlation and used as input to the Cytoscape program where, in addition to visual representations, measures of betweenness centrality were obtained.

| **Taxon** | **Betw. cent.** | **Taxon** | **Betw. cent.** | **Taxon** | **Betw. cent.** |
| --- | --- | --- | --- | --- | --- |
| *Nitrosospira* | 0.0848 | *Marmoricola* | 0.025 | *Flavitalea* | 0.0093 |
| *Ktedonobacter* | 0.0754 | *Kaistia* | 0.0249 | *Mucilaginibacter* | 0.0092 |
| *Kribbella* | 0.0735 | *Myxococcus* | 0.0247 | *Luteibacter* | 0.0092 |
| *Burkholderia* | 0.0662 | *Polyangium* | 0.0245 | *Micromonospora* | 0.0087 |
| *Nitrospira* | 0.0624 | *Flavobacterium* | 0.0243 | *Streptomyces* | 0.0085 |
| *Phenylobacterium* | 0.061 | *Domibacillus* | 0.0243 | *Dokdonella* | 0.0084 |
| *Skermanella* | 0.0607 | *Niastella* | 0.0231 | *Chitinophaga* | 0.0082 |
| *Parasegetibacter* | 0.0545 | *Methylobacterium* | 0.0217 | *Aciditerrimonas* | 0.0078 |
| *Jatrophihabitans* | 0.0543 | *Paenibacillus* | 0.0208 | *Gemmatimonas* | 0.0073 |
| *Aquicella* | 0.0527 | *Rhizobium* | 0.0195 | *Minicystis* | 0.0069 |
| *Conexibacter* | 0.0508 | *Flavisolibacter* | 0.0194 | *Nonomuraea* | 0.0069 |
| *Mesorhizobium* | 0.0494 | *Janibacter* | 0.0188 | *Agromyces* | 0.0066 |
| *Dactylosporangium* | 0.0479 | *Duganella* | 0.0188 | *Bradyrhizobium* | 0.0061 |
| *Povalibacter* | 0.0478 | *Sorangium* | 0.0182 | *Sinomonas* | 0.0055 |
| *Sandaracinus* | 0.046 | *Opitutus* | 0.0181 | *Ramlibacter* | 0.0055 |
| *Pseudonocardia* | 0.0441 | *Aggregicoccus* | 0.0177 | *Virgisporangium* | 0.0054 |
| *Segetibacter* | 0.0411 | *Nocardioides* | 0.0173 | *Alsobacter* | 0.0054 |
| *Bacillus* | 0.0405 | *Terrimonas* | 0.0171 | *Nannocystis* | 0.0054 |
| *Arenimonas* | 0.0384 | *Pseudomonas* | 0.0156 | *Enterobacter* | 0.0045 |
| *Tumebacillus* | 0.0374 | *Alicyclobacillus* | 0.0155 | *Spirosoma* | 0.0024 |
| *Mycobacterium* | 0.0373 | *Catenulispora* | 0.0154 | *Kofleria* | 0.0023 |
| *Solirubrobacter* | 0.0359 | *Variovorax* | 0.0145 | *Noviherbaspirillum* | 0.0021 |
| *Nakamurella* | 0.0358 | *Intrasporangium* | 0.0143 | *Rugosimonospora* | 0.001 |
| *Kitasatospora* | 0.0342 | *Amycolatopsis* | 0.0143 | *Blastococcus* | 0.0008 |
| *Devosia* | 0.0327 | *Geodermatophilus* | 0.0137 | *Arthrobacter* | 0.0007 |
| *Phaselicystis* | 0.0325 | *Herminiimonas* | 0.0135 | *Labilithrix* | 0.0001 |
| *Hyphomicrobium* | 0.0319 | *Anaeromyxobacter* | 0.0134 | *Porphyrobacter* | 0 |
| *Acinetobacter* | 0.0319 | *Stenotrophomonas* | 0.0132 | *Oxalophagus* | 0 |
| *Gaiella* | 0.0304 | *Labrys* | 0.0132 | *Terrabacter* | 0 |
| *Rubellimicrobium* | 0.0304 | *Sphingomonas* | 0.0131 | *Ilumatobacter* | 0 |
| *Pelomonas* | 0.0303 | *Leifsonia* | 0.0131 | *Cystobacter* | 0 |
| *Massilia* | 0.0299 | *Angustibacter* | 0.0131 | *Byssovorax* | 0 |
| *Pedomicrobium* | 0.0287 | *Cupriavidus* | 0.0128 | *Dyella* | 0 |
| *Lysobacter* | 0.0287 | *Clostridium_sensu_stricto* | 0.0121 | *Actinospica* | 0 |
| *Phycicoccus* | 0.0286 | *Luteimonas* | 0.0121 | *Caldilinea* | 0 |
| *Microvirga* | 0.0264 | *Dongia* | 0.012 | *-* | - |
| *Roseiarcus* | 0.0259 | *Luteolibacter* | 0.0111 | *-* | - |

**Supplementary Table 11:** Fungal genera identified through Pearson's correlation and used as input to the Cytoscape program where, in addition to visual representations, the numbers of correlations (positive and negative) established between the genera were obtained.

| **Taxon** | **Corr.** | **Taxon** | **Corr.** | **Taxon** | **Corr.** |
| --- | --- | --- | --- | --- | --- |
| *Ascobolus* | 27 | *Chaetosphaeria* | 18 | *Cylindrocarpon* | 10 |
| *Microdochium* | 26 | *Chaetomium* | 18 | *Pseudocoleophoma* | 10 |
| *Macrophomina* | 26 | *Veronaea* | 18 | *Parazalerion* | 10 |
| *Phialophora* | 25 | *Ochroconis* | 18 | *Spegazzinia* | 9 |
| *Cyphellophora* | 25 | *Rhytidhysteron* | 18 | *Humicola* | 9 |
| *Sporothrix* | 25 | *Trechispora* | 18 | *Phialemoniopsis* | 9 |
| *Mycothermus* | 25 | *Clitopilus* | 18 | *Achroiostachys* | 9 |
| *Acrocalymma* | 25 | *Beauveria* | 18 | *Alfaria* | 9 |
| *Polylobatispora* | 24 | *Gongronella* | 18 | *Pseudoramichloridium* | 9 |
| *Basidioascus* | 24 | *Sistotrema* | 18 | *Tubeufia* | 9 |
| *Coniocessia* | 24 | *Oidiodendron* | 18 | *Trichosporon* | 9 |
| *Ijuhya* | 24 | *Cladorrhinum* | 17 | *Absidia* | 9 |
| *Preussia* | 24 | *Resinicium* | 17 | *Cladophialophora* | 8 |
| *Colletotrichum* | 24 | *Fluminicola* | 17 | *Serendipita* | 8 |
| *Dactylella* | 24 | *Periconia* | 17 | *Bipolaris* | 8 |
| *Subramaniula* | 24 | *Neodevriesia* | 17 | *Stagonospora* | 8 |
| *Psathyrella* | 23 | *Lycoperdon* | 17 | *Debaryomyces* | 8 |
| *Byssochlamys* | 23 | *Podospora* | 16 | *Ramichloridium* | 8 |
| *Strelitziana* | 23 | *Saitozyma* | 16 | *Atractiella* | 8 |
| *Talaromyces* | 23 | *Cuniculitrema* | 16 | *Rhizophagus* | 8 |
| *Agrocybe* | 23 | *Myrmecridium* | 16 | *Xenoacremonium* | 8 |
| *Phaeosphaeria* | 23 | *Helicogloea* | 16 | *Exophiala* | 8 |
| *Pseudopithomyces* | 23 | *Metarhizium* | 16 | *Dichotomopilus* | 8 |
| *Tetracladium* | 23 | *Vermispora* | 16 | *Corymbiglomus* | 8 |
| *Phialocephala* | 23 | *Staphylotrichum* | 15 | *Exserohilum* | 8 |
| *Fusariella* | 23 | *Chloridium* | 15 | *Nectriopsis* | 8 |
| *Pyrenochaeta* | 23 | *Kockovaella* | 15 | *Arthrobotrys* | 8 |
| *Zopfiella* | 23 | *Immersiella* | 15 | *Cladosporium* | 7 |
| *Acremonium* | 23 | *Scopulariopsis* | 14 | *Hannaella* | 7 |
| *Nothopassalora* | 23 | *Fusicolla* | 14 | *Paracremonium* | 7 |
| *Claroideoglomus* | 23 | *Aspergillus* | 14 | *Kendrickiella* | 7 |
| *Coprinopsis* | 22 | *Fusarium* | 14 | *Nigrospora* | 7 |
| *Epicoccum* | 22 | *Westerdykella* | 14 | *Lectera* | 6 |
| *Bjerkandera* | 22 | *Albifimbria* | 13 | *Xepicula* | 6 |
| *Paratrimmatostroma* | 22 | *Antennariella* | 13 | *Mortierella* | 6 |
| *Aureobasidium* | 22 | *Xenasmatella* | 13 | *Xenomyrothecium* | 6 |
| *Synnemellisia* | 22 | *Remersonia* | 13 | *Pyrenochaetopsis* | 6 |
| *Cirrenalia* | 22 | *Xenopenidiella* | 13 | *Cetraspora* | 6 |
| *Trichoderma* | 22 | *Keissleriella* | 12 | *Setophoma* | 5 |
| *Clonostachys* | 22 | *Apiotrichum* | 12 | *Conioscypha* | 5 |
| *Spizellomyces* | 22 | *Penicillium* | 12 | *Zygosporium* | 5 |
| *Solicoccozyma* | 22 | *Helicoma* | 12 | *Glomerella* | 5 |
| *Clavulinopsis* | 22 | *Arxiella* | 12 | *Acaulospora* | 5 |
| *Codinaea* | 21 | *Sarocladium* | 12 | *Dictyosporium* | 5 |
| *Naganishia* | 21 | *Hormonema* | 12 | *Hansfordia* | 5 |
| *Striaticonidium* | 21 | *Pluteus* | 12 | *Papiliotrema* | 4 |
| *Microascus* | 21 | *Chordomyces* | 11 | *Scutellinia* | 4 |
| *Acrodontium* | 21 | *Penicillifer* | 11 | *Sakaguchia* | 4 |
| *Rhizoctonia* | 21 | *Escovopsis* | 11 | *Scytalidium* | 4 |
| *Hyphoderma* | 21 | *Occultifur* | 11 | *Paraphaeosphaeria* | 4 |
| *Coprinellus* | 21 | *Conlarium* | 11 | *Plectosphaerella* | 4 |
| *Neoidriella* | 20 | *Lipomyces* | 11 | *Tetraplosphaeria* | 3 |
| *Panaeolus* | 20 | *Monocillium* | 11 | *Papulaspora* | 3 |
| *Neurospora* | 20 | *Cutaneotrichosporon* | 11 | *Paecilomyces* | 3 |
| *Pseudorobillarda* | 20 | *Hypomyces* | 11 | *Arcopilus* | 3 |
| *Conocybe* | 20 | *Curvularia* | 11 | *Roussoella* | 3 |
| *Sagenomella* | 19 | *Stachylidium* | 11 | *Mariannaea* | 2 |
| *Alternaria* | 19 | *Leuconeurospora* | 11 | *Gibellulopsis* | 2 |
| *Tremella* | 19 | *Torulaspora* | 10 | *Coniochaeta* | 2 |
| *Paraglomus* | 19 | *Chrysosporium* | 10 | *Rhodosporidiobolus* | 1 |
| *Ceratocystis* | 19 | *Paraconiothyrium* | 10 | *Coniophora* | 1 |
| *Metacordyceps* | 19 | *Rhexoacrodictys* | 10 | *-* | - |
| *Rhizopus* | 18 | *Torula* | 10 | *-* | - |

**Supplementary Table 12:** Fungal genera identified through Pearson's correlation and used as input to the Cytoscape program where, in addition to visual representations, measures of betweenness centrality were obtained.

| **Taxon** | **Betw. cent.** | **Taxon** | **Betw. cent.** | **Taxon** | **Betw. cent.** |
| --- | --- | --- | --- | --- | --- |
| *Rhizopus* | 0.0623 | *Cuniculitrema* | 0.0165 | *Corymbiglomus* | 0.0065 |
| *Scopulariopsis* | 0.0471 | *Antennariella* | 0.0165 | *Xenasmatella* | 0.0065 |
| *Podospora* | 0.0454 | *Panaeolus* | 0.0165 | *Remersonia* | 0.0065 |
| *Cladophialophora* | 0.045 | *Tetraplosphaeria* | 0.0165 | *Aureobasidium* | 0.0065 |
| *Keissleriella* | 0.0442 | *Tremella* | 0.0164 | *Metarhizium* | 0.0064 |
| *Serendipita* | 0.0436 | *Helicoma* | 0.0161 | *Preussia* | 0.0063 |
| *Chordomyces* | 0.0432 | *Paracremonium* | 0.0161 | *Papulaspora* | 0.0062 |
| *Spegazzinia* | 0.0421 | *Glomerella* | 0.016 | *Curvularia* | 0.0061 |
| *Fusicolla* | 0.0408 | *Bjerkandera* | 0.0152 | *Phialocephala* | 0.0061 |
| *Chaetosphaeria* | 0.0402 | *Basidioascus* | 0.0146 | *Naganishia* | 0.0061 |
| *Chaetomium* | 0.039 | *Byssochlamys* | 0.014 | *Microdochium* | 0.0059 |
| *Penicillifer* | 0.039 | *Kendrickiella* | 0.0138 | *Macrophomina* | 0.0059 |
| *Staphylotrichum* | 0.0388 | *Scutellinia* | 0.0136 | *Phialophora* | 0.0058 |
| *Aspergillus* | 0.036 | *Strelitziana* | 0.0132 | *Cyphellophora* | 0.0058 |
| *Neoidriella* | 0.0358 | *Ochroconis* | 0.013 | *Rhexoacrodictys* | 0.0056 |
| *Coprinopsis* | 0.0355 | *Arxiella* | 0.0128 | *Paecilomyces* | 0.0054 |
| *Cladosporium* | 0.0338 | *Sakaguchia* | 0.0126 | *Fusariella* | 0.0051 |
| *Fusarium* | 0.0318 | *Resinicium* | 0.0123 | *Nigrospora* | 0.005 |
| *Bipolaris* | 0.0313 | *Talaromyces* | 0.0121 | *Exserohilum* | 0.005 |
| *Humicola* | 0.0304 | *Tubeufia* | 0.0119 | *Clitopilus* | 0.0049 |
| *Escovopsis* | 0.0301 | *Acaulospora* | 0.0118 | *Beauveria* | 0.0049 |
| *Saitozyma* | 0.0297 | *Agrocybe* | 0.0117 | *Colletotrichum* | 0.0049 |
| *Phialemoniopsis* | 0.029 | *Dictyosporium* | 0.0116 | *Synnemellisia* | 0.0048 |
| *Stagonospora* | 0.0283 | *Mortierella* | 0.0116 | *Cirrenalia* | 0.0047 |
| *Westerdykella* | 0.0275 | *Lipomyces* | 0.0114 | *Trichoderma* | 0.0046 |
| *Setophoma* | 0.0263 | *Fluminicola* | 0.0112 | *Hormonema* | 0.0045 |
| *Epicoccum* | 0.026 | *Chloridium* | 0.0108 | *Clonostachys* | 0.0044 |
| *Torulaspora* | 0.0259 | *Phaeosphaeria* | 0.0108 | *Nectriopsis* | 0.0044 |
| *Conioscypha* | 0.0253 | *Xenomyrothecium* | 0.0108 | *Plectosphaerella* | 0.0043 |
| *Polylobatispora* | 0.0252 | *Trichosporon* | 0.0107 | *Pyrenochaeta* | 0.0043 |
| *Lectera* | 0.0249 | *Rhytidhysteron* | 0.0102 | *Arcopilus* | 0.0042 |
| *Achroiostachys* | 0.0243 | *Myrmecridium* | 0.0099 | *Torula* | 0.0041 |
| *Papiliotrema* | 0.0242 | *Pyrenochaetopsis* | 0.0097 | *Sporothrix* | 0.004 |
| *Alfaria* | 0.024 | *Exophiala* | 0.0094 | *Mycothermus* | 0.004 |
| *Occultifur* | 0.0239 | *Absidia* | 0.0091 | *Acrocalymma* | 0.004 |
| *Debaryomyces* | 0.0238 | *Chrysosporium* | 0.0091 | *Periconia* | 0.0037 |
| *Ramichloridium* | 0.0232 | *Scytalidium* | 0.0088 | *Dactylella* | 0.0035 |
| *Apiotrichum* | 0.023 | *Paraglomus* | 0.0087 | *Neurospora* | 0.0034 |
| *Zygosporium* | 0.0228 | *Monocillium* | 0.0085 | *Zopfiella* | 0.0032 |
| *Veronaea* | 0.0223 | *Sarocladium* | 0.0085 | *Arthrobotrys* | 0.0031 |
| *Atractiella* | 0.022 | *Dichotomopilus* | 0.0084 | *Cylindrocarpon* | 0.0031 |
| *Sagenomella* | 0.0213 | *Pseudopithomyces* | 0.0082 | *Pluteus* | 0.0031 |
| *Cladorrhinum* | 0.0202 | *Paraphaeosphaeria* | 0.0082 | *Subramaniula* | 0.003 |
| *Hannaella* | 0.0199 | *Paraconiothyrium* | 0.0081 | *Gongronella* | 0.0029 |
| *Pseudoramichloridium* | 0.0199 | *Tetracladium* | 0.0078 | *Pseudocoleophoma* | 0.0028 |
| *Alternaria* | 0.0194 | *Coniocessia* | 0.0077 | *Parazalerion* | 0.0028 |
| *Rhizophagus* | 0.0188 | *Kockovaella* | 0.0076 | *Sistotrema* | 0.0028 |
| *Ascobolus* | 0.0184 | *Ijuhya* | 0.0074 | *Oidiodendron* | 0.0028 |
| *Codinaea* | 0.0183 | *Cutaneotrichosporon* | 0.0073 | *Striaticonidium* | 0.0026 |
| *Psathyrella* | 0.0179 | *Helicogloea* | 0.0073 | *Acremonium* | 0.0026 |
| *Xenoacremonium* | 0.0178 | *Hypomyces* | 0.0072 | *Cetraspora* | 0.0025 |
| *Conlarium* | 0.0177 | *Trechispora* | 0.007 | *Hansfordia* | 0.0025 |
| *Albifimbria* | 0.0175 | *Paratrimmatostroma* | 0.0069 | *Microascus* | 0.0023 |
| *Xepicula* | 0.0172 | *Mariannaea* | 0.0066 | *-* | - |
| *Penicillium* | 0.0171 | *Ceratocystis* | 0.0066 | *-* | - |
